# Enhancing insights into diseases through horizontal gene transfer event detection from gut microbiome

**DOI:** 10.1093/nar/gkae515

**Published:** 2024-06-17

**Authors:** Shuai Wang, Yiqi Jiang, Lijia Che, Ruo Han Wang, Shuai Cheng Li

**Affiliations:** City University of Hong Kong Shenzhen Research Institute, Shenzhen, China; Department of Computer Science, City University of Hong Kong, Kowloon, Hong Kong; City University of Hong Kong Shenzhen Research Institute, Shenzhen, China; Department of Computer Science, City University of Hong Kong, Kowloon, Hong Kong; City University of Hong Kong Shenzhen Research Institute, Shenzhen, China; Department of Computer Science, City University of Hong Kong, Kowloon, Hong Kong; City University of Hong Kong Shenzhen Research Institute, Shenzhen, China; Department of Computer Science, City University of Hong Kong, Kowloon, Hong Kong; City University of Hong Kong Shenzhen Research Institute, Shenzhen, China; Department of Computer Science, City University of Hong Kong, Kowloon, Hong Kong

## Abstract

Horizontal gene transfer (HGT) phenomena pervade the gut microbiome and significantly impact human health. Yet, no current method can accurately identify complete HGT events, including the transferred sequence and the associated deletion and insertion breakpoints from shotgun metagenomic data. Here, we develop LocalHGT, which facilitates the reliable and swift detection of complete HGT events from shotgun metagenomic data, delivering an accuracy of 99.4%—verified by Nanopore data—across 200 gut microbiome samples, and achieving an average F1 score of 0.99 on 100 simulated data. LocalHGT enables a systematic characterization of HGT events within the human gut microbiome across 2098 samples, revealing that multiple recipient genome sites can become targets of a transferred sequence, microhomology is enriched in HGT breakpoint junctions (*P*-value = 3.3e-58), and HGTs can function as host-specific fingerprints indicated by the significantly higher HGT similarity of intra-personal temporal samples than inter-personal samples (*P*-value = 4.3e-303). Crucially, HGTs showed potential contributions to colorectal cancer (CRC) and acute diarrhoea, as evidenced by the enrichment of the butyrate metabolism pathway (*P*-value = 3.8e-17) and the shigellosis pathway (*P*-value = 5.9e-13) in the respective associated HGTs. Furthermore, differential HGTs demonstrated promise as biomarkers for predicting various diseases. Integrating HGTs into a CRC prediction model achieved an AUC of 0.87.

## Graphical Abstract

**Graphical Abstract:**
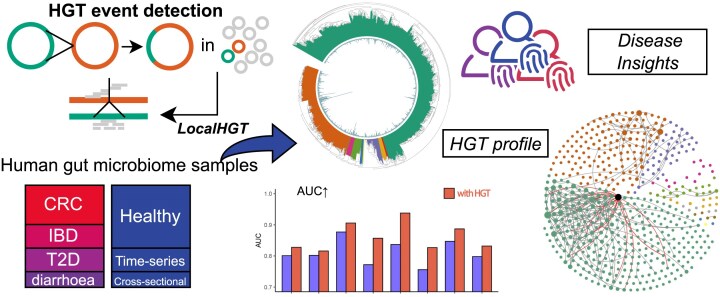

## Introduction

Horizontal gene transfer (HGT) refers to the transmission of genetic materials between organisms and is recognized as an essential factor in microbial evolution and adaptation (1,2). Studies have shown that bacteriophage-mediated HGT is more vital than mutation for *Escherichia coli* to adapt in the mammalian gut microbiome (3). HGT can facilitate the spread of virulence factors among bacteria (4,5). For instance, the highly virulent strain *E. coli* O104:H4, which caused the outbreak of diarrhea and the hemolytic–uremic syndrome in Germany, emerged due to an HGT event (6,7). Moreover, HGTs can accelerate the global dissemination of antibiotic resistance genes (ARGs) among microbes (4,8,9). For example, *Staphylococcus aureus* acquired the vancomycin-resistant gene from *Enterococcus faecalis* through HGT (10,11). In another instance, the transfer of a single azithromycin resistance plasmid contributed to the outbreak of multiple *Shigella* species in the United Kingdom (12). The complex gut ecosystem harbors a large and diverse bacterial population, offering abundant opportunities for HGT events (13–15), which are critical for the human health. For example, as the gut microbiome is a major reservoir of ARGs, bacterial pathogens in the gut microbiome can potentially acquire ARGs from nonpathogens via HGT, driving the evolution of pathogens resistant to antibiotics (16–18). In contrast, it is proposed that *Lachnospiraceae* members acquire butyric acid production functions through HGT, thereby protecting human against colorectal cancer (CRC) (19).

Numerous methods have been developed to detect HGTs based on isolate genomes. However, these methods are not suitable for characterizing HGTs within the gut microbiome (20). Recently, several studies have attempted to analyze HGTs within the gut microbiome. Groussin *et al.* collected over 4000 isolated and sequenced gut bacteria from diverse populations, and detected HGTs by screening identical DNA blocks between the bacteria assemblies. Using this approach, they found that HGTs occur frequently in the gut microbiome of individuals, with a higher occurrence rate observed in industrialized and urban populations (14). However, this approach requires expensive and laborious efforts, and it is unable to detect HGTs of uncultured bacteria. Some researchers aimed to characterize structural variations (SVs) within the human gut microbiome. SVs are defined as genome segments that exist in varying copy numbers across different individuals. They discovered that SVs are associated with microbial adaptation, host lifestyles, and host disease risk factors (21–23). Since one of the primary driving forces of SV could be HGT (21), these SV analyses indirectly demonstrate the association between HGTs and host health. Nevertheless, these analyses cannot distinguish within-cell variations and HGTs, limiting their application for the understanding of HGTs. Recently, some studies directly identify HGTs from shotgun metagenomic data by performing metagenomic assembly or aligning sequencing reads to the reference database. MetaCHIP adopts both best-match and phylogenetic approaches to deduce HGTs based on the assembled contigs (20). DaisySuite and LEMON analyze sequencing reads aligned to the reference database to infer HGTs. DaisySuite identifies HGT recipient and donor genomes based on read coverage and determines HGT boundaries using split-reads (24,25). LEMON, on the other hand, infers HGT breakpoints using the DBSCAN clustering algorithm with junction reads (26). Nevertheless, the widespread adoption of these methods has been limited, likely due to the high computational resource requirements and the inconvenience of installation associated with these methods. Moreover, it is crucial to deduce complete HGT events, encompassing the transferred sequence as well as the corresponding deletion and insertion sites in the donor and recipient genomes, respectively. Deducing complete HGT events not only allows us to study the detailed transfer patterns of HGTs, but also enables us to gain a comprehensive understanding of the functions of HGTs. Furthermore, it provides an opportunity to delve into the underlying mutational mechanisms of HGTs. However, currently, there is no reliable method to deduce complete HGT events.

The *k*-mer technique is widely employed in metagenomics to minimize computational resource consumption. For instance, Kraken utilizes exact alignment of *k*-mers instead of read alignment for metagenomic taxonomic classification (27), while Metalign employs containment min hash and *k*-mers to predict the presence and abundances of microbes (28). Also, GT-Pro utilizes *k*-mers to rapidly genotype SNPs in metagenomic samples by uniquely probing the allele of each SNP (29). These approaches have inspired us to leverage *k*-mers for accelerated detection of HGTs in metagenomic data. However, these methods rely on exact matching of *k*-mers, making them susceptible to sequencing errors and genetic variations (30). To address this challenge, we have developed a novel *k*-mer encoding algorithm to achieve *fuzzy k-mer matching*. This involves allocating an identical hash value to similar *k*-mers by using multiple hash functions.

In this study, we present LocalHGT, a user-friendly software package for reliable and efficient detection of complete HGT events from shotgun metagenomic data. LocalHGT utilizes fast fuzzy *k*-mer matching to identify *HGT-related segments*, i.e., reference segments potentially containing HGT breakpoints, from a comprehensive reference database. It subsequently detects precise HGT breakpoints by mapping sequencing reads to these HGT-related segments. Finally, it matches the identified HGT breakpoint pairs to deduce complete HGT events. The package demonstrated reliable performance, achieving a 99.4% accuracy in determining complete HGT events across 200 shotgun metagenomic samples. This accuracy was assessed with matched Nanopore long-read sequencing data. When tested on 100 simulated data, it achieved an F1 score of 0.99. Additionally, it demonstrated significant computational advantages over the traditional alignment-based tool. On average, it required 82.7% less CPU time in handling gut microbiome data. Leveraging LocalHGT, we systematically analyzed HGTs within the human gut microbiome for 2,098 samples from various diseases, including CRC, adenoma, type 2 diabetes (T2D), impaired glucose tolerance (IGT), acute diarrhea, and inflammatory bowel disease (IBD). First, the HGT events identified by LocalHGT revealed that multiple recipient genome sites can become targets of a transferred sequence. Non-surprisingly, the frequency of HGT is negatively correlated with the phylogenetic distance between the genomes involved. Notably, the identified HGT events showed significant enrichment of microhomology in HGT breakpoint junctions, with nonhomologous end joining (NHEJ) and alternative end joining (alt-EJ) emerging as the primary mutational mechanisms driving HGT formation. Furthermore, these HGT events suggested that they can function as host-specific fingerprints, due to their time-stability and person-specific nature. The functional analysis unveiled the pivotal role of HGTs in microbial adaptation, as indicated by their significant enrichment in bacterial defense mechanisms and secretion functions. Significantly, these HGT events have demonstrated their potential contributions to CRC and acute diarrhoea. This is supported by the notable enrichment of the butyrate metabolism pathway (*P*-value = 3.8e-17) and the shigellosis pathway (entry: hsa05131), which represents the mechanism by which the *Shigella* bacteria infect human intestinal cells (*P*-value = 5.9e-13), in the associated HGTs for each respective disease. Furthermore, the differential HGT events demonstrated their utility as biomarkers for predicting various diseases. Differential HGTs between individuals with the disease and healthy individuals served as disease-specific biomarkers, with the presence or absence of the HGTs representing biomarker values. Utilizing HGTs solely achieved high area under the curve (AUC) values for predicting CRC (0.82), IGT (0.91), acute diarrhea (0.98) and IBD (0.79). Integrating HGTs in a CRC prediction model resulted in an AUC of 0.87, surpassing the performance of using solely microbial-abundance biomarkers (0.81). Additionally, the HGT network formed by HGT events exhibited species associated with human diseases. Collectively, LocalHGT enables systematic analysis of HGTs within the microbiome, highlighting its utility for improving insights into diseases.

## Materials and methods

### Algorithm of LocalHGT

#### Encoding of *k*-mers

**Figure 1.**
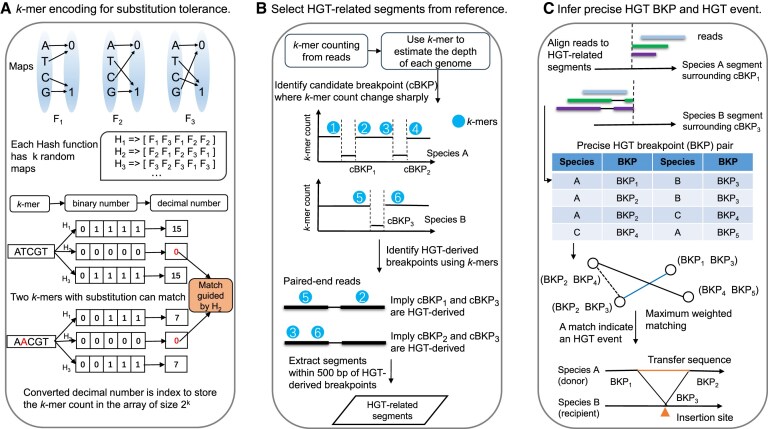
Workflow of the HGT detection method. (**A**) Illustration of the *k*-mer encoding method. We construct three maps to encode the DNA bases into binary digits. Each map assigns the same value (0 or 1) to every two distinct bases. We then create several hash functions, each consisting of *k* random maps. Given a *k*-mer, we encode it into a binary string and convert it to a decimal number using each hash function. Each *k*-mer has multiple encoded integers, representing the index to store its count in the array. This approach allows two *k*-mers with substitutions to be encoded to the same value. (**B**) Identification of HGT-related reference segments potentially containing HGT breakpoints. First, we perform *k*-mer counting from sequencing reads. Then, we enumerate each *k*-mer along the reference genomes and select loci with sharp *k*-mer count changes, referred to as cBKPs. The *k*-mer at each cBKP locus is chosen as markers (blue circle symbol). Markers originating from different species but aligning to the same paired-end read indicate HGT-derived cBKPs. Reference segments surrounding the HGT-derived cBKP loci are extracted as HGT-related segments. (**C**) Precise HGT breakpoint detection and HGT event inference. The sequencing reads are mapped to the HGT-related segments using BWA MEM. The precise HGT breakpoints are obtained based on junction reads. Subsequently, each HGT breakpoint pair is represented as a node, and an edge between two nodes indicates the potential formation of an HGT event. We employ maximum weighted matching to infer complete HGT events. For each HGT event, we identify the transferred sequence from the donor genome and the insertion site on the recipient genome.

Fuzzy *k*-mer matching, enabling the matching of similar *k*-mers, is achieved using a novel *k*-mer encoding algorithm (Figure 1A and Supplementary Figure S1). This algorithm involves the construction of three maps that facilitate the encoding of DNA bases into binary digits within the *k*-mer. DNA sequences are composed of four distinct types of bases, namely A, T, C and G. When comparing two DNA sequences, there exist up to 12 potential types of substitutions, including A>T, T>A, C>G and others. To effectively tolerate substitutions in *k*-mer matching, three different maps are introduced, where each map assigns a consistent value (0 or 1) to every pair of distinct bases. The maps are defined as


(1) \begin{eqnarray*}
\begin{aligned} F_1(x)=\lbrace 0:x \in \lbrace A,T\rbrace , 1:x \in \lbrace C,G\rbrace \rbrace , \\ F_2(x)=\lbrace 0:x \in \lbrace A,C\rbrace , 1:x \in \lbrace T,G\rbrace \rbrace , \\ F_3(x)=\lbrace 0:x \in \lbrace A,G\rbrace , 1:x \in \lbrace T,C\rbrace \rbrace . \end{aligned}
\end{eqnarray*}

This encoding scheme allows the tolerance of four types of substitutions in each map and enables the tolerance of every substitution at a locus with all three maps.

For encoding the *k*-mer, a hash function denoted as *H* is constructed by utilizing *k* random maps. The hash function *H* incorporates an array of *k* random maps (Φ), where Φ_*j*_ ∈ {*F*_1_, *F*_2_, *F*_3_} and 1 ≤ *j* ≤ *k*. Given a *k*-mer represented as α = (α_1_α_2_...α_*k*_), where α_*j*_ ∈ {*A*, *T*, *C*, *G*}, the *k*-mer is converted into a binary number β = (β_1_β_2_...β_*k*_) through the application of the mapping function β_*j*_ = Φ_*j*_(α_*j*_). Next, the hash function *H* further transforms the binary number β into an integer δ using the formula


(2) \begin{eqnarray*}
\delta = \sum _{j=1}^k \beta _j 2^j.
\end{eqnarray*}

The reverse complementary sequence of the given *k*-mer α is denoted as $\bar{\alpha }$. Utilizing the same encoding approach, $\bar{\alpha }$ is converted into an integer $\bar{\delta }$. The encoded value for the given *k*-mer α is determined as $H(\alpha ) = \min \lbrace \delta , \bar{\delta }\rbrace$.

To improve the substitution tolerance, each *k*-mer is encoded using ℓ hash functions (ℓ = 3 by default). The assignment of the combinatorial maps to the hash functions at each locus of the *k*-mer is accomplished through the following procedure. Considering the three maps (*F*_1_, *F*_2_, *F*_3_), there are 3! = 6 possible permutations. At locus *j* of the *k*-mer, we randomly select ⌈ℓ/3⌉ permutations and concatenate them into an array *P*. Here, *P*_*i*_ ∈ {*F*_1_, *F*_2_, *F*_3_} represents the map used by the *i*-th hash function *H*_*i*_ at locus *j*.

The encoded integers of each *k*-mer is used to index the hash table. Each *k*-mer is encoded by ℓ hash functions, resulting in ℓ integers. The *k*-mers are encoded using the same set of hash functions in all steps of LocalHGT. To store the counts of *k*-mers, an array denoted as *Q* is utilized, serving as the hash table. The size of the hash table is set to 2^*k*^. Each *k*-mer is stored ℓ times within the hash table, using the encoded integers as indices. It is possible for hash collisions to occur, meaning that different *k*-mers may be mapped to the same hash value. However, our scheme of the hash function manages the occurrences of collisions well (Supplementary Note S1 and Supplementary Figure S2). The elements in the array *Q* use the ‘byte’ datatype, and each hash table consumes 2^*k*^ bytes of memory. The default value for *k* is 32.

#### Extraction of HGT-related segments

To reduce computational resources for aligning reads to a large comprehensive reference database, a fast fuzzy *k*-mer matching technique extracts HGT-related segments from the reference database. These segments are reference segments potentially containing HGT breakpoints. If a sequencing read covers the junction of an HGT breakpoint, it will be mapped to two distinct species genomes or distant genome loci. This characteristic pattern is utilized to identify the HGT breakpoint junctions. Subsequently, the genomic segments surrounding these junctions are extracted as HGT-related segments.

To conduct *k*-mer counting from the sequencing reads, the aforementioned *k*-mer encoding method is employed (Figure 1B). To mitigate the likelihood of hash collisions, downsampling of the sequencing reads is performed during the *k*-mer counting process. The selection of reads is conducted randomly using a sampling rate of *M*/*A*, where *A* represents the total number of DNA bases across all reads in a given sample, and *M* is a hyper-parameter with a default value of *M* = 2 × 10^9^. The *k*-mers present in the selected reads are enumerated, encoded into integers, and utilized as indices in the hash table *Q* to store their respective counts. The presence of each *k*-mer leads to an increment of one in all the indexed values within *Q*.

Afterward, reference fragments that may be present in the given sample are extracted using *k*-mer matching. The *k*-mers along the reference genome are enumerated. Each *k*-mer on the reference is encoded into ℓ integers using the same hash functions employed in *k*-mer counting. These integers are used to access the corresponding count values *q*_1_, *q*_2_, ..., *q*_ℓ_ in the hash table *Q*. Boolean variables *E* and *Z* are utilized to indicate whether a *k*-mer generates an exact hit and a fuzzy hit, respectively. With a threshold value *t* (default: 3), *E* and *Z* are determined by


(3) \begin{eqnarray*}
E = \left\lbrace \begin{array}{@{}l@{\quad }l@{}}1, & \text{if } \min (q_1, q_2, ..., q_\ell ) \ge t \\ 0, & \text{otherwise} \end{array}\right.
\end{eqnarray*}

(4) \begin{eqnarray*}
Z = \left\lbrace \begin{array}{@{}l@{\quad }l@{}}1, & \text{if } \max (q_1, q_2, ..., q_\ell ) \ge t \\ 0, & \text{otherwise.} \end{array}\right.
\end{eqnarray*}

Fragments that are present in the given sample typically demonstrate a higher number of *k*-mer hits compared to fragments that are absent. To extract these fragments, a window-sliding technique is employed. Suppose the length of the window is *w* (default: 500), and the number of *k*-mers in the window is *u* = *w* − *k* + 1. The exact hit ratio (κ) in a window is defined as


(5) \begin{eqnarray*}
\kappa =\sum _{r=1}^{u}\frac{E_r}{u},
\end{eqnarray*}

where *r* is the index of the *k*-mer in the window. Similarly, the fuzzy hit ratio (μ) in a window is calculated as


(6) \begin{eqnarray*}
\mu =\sum _{r=1}^{u}\frac{Z_r}{u}.
\end{eqnarray*}

Fragments within windows satisfying the conditions of κ ≥ *m*_1_ and μ ≥ *m*_2_ are designated as the fragments present in the given sample (*m*_1_ = 0.08 and *m*_2_ = 0.1 by default). Conversely, the remaining fragments are excluded from subsequent procedures.

By utilizing *k*-mer counts, candidate breakpoint (cBKP) loci characterized by abrupt changes in *k*-mer count along the reference genome are identified. For each *k*-mer on the reference, its count is determined as *D* = max (*q*_1_, *q*_2_, ..., *q*_ℓ_). The corresponding *k*-mer for a given locus *z* is defined as the sequence spanning from position *z* to *z* + *k* − 1 on the reference genome. Loci are examined to identify instances where the counts of their corresponding *k*-mers exhibit pronounced changes. To ensure robust analysis, we compute the average count of *k*-mers within a bin of length *e* (default: 5). The average *k*-mer count (ξ_*z*_) for a bin starting at locus *z* is calculated as:


(7) \begin{eqnarray*}
\xi _z = \sum _{i=z}^{z+e-1} \frac{D_i}{e},
\end{eqnarray*}

where *D*_*i*_ represents the count of the *k*-mer at locus *i*. Comparisons are then made between the average counts of each bin and the adjacent bins. Considering a bin with the start locus at *z*, we compare it with *k* subsequent bins, with their start loci ranging from *z* + *e* to *z* + *e* + *k*. The count difference between bin *z* and each following bin *d* is calculated as:


(8) \begin{eqnarray*}
\epsilon = \xi _z - \xi _d.
\end{eqnarray*}

If ε exceeds a positive cutoff threshold θ (default: 3), the locus *z* is selected as a cBKP locus. Conversely, if ε is smaller than −θ, the locus *d* is chosen as a cBKP locus.

Next, we select HGT-derived cBKP loci, which are cBKP loci located on HGT breakpoint junctions. Mapping a sequencing read to two cBKPs from distinct species genomes suggests that the read originates from an HGT breakpoint junction, with both cBKPs being HGT-derived. This pattern is utilized to identify HGT-derived cBKP loci. For each cBKP locus *p*, we extract its corresponding *k*-mer from the reference sequence as its marker. The array *B* is utilized to store the relationship between each marker *k*-mer and its corresponding cBKP locus *p*. The marker *k*-mer is encoded using the aforementioned hash functions, and the resulting encoded integers are used as indices to access and store the corresponding cBKP locus *p* in the array *B*. The elements in the hash table *B* use the ‘int’ datatype and *B* consumes 4 × 2^*k*^ bytes of memory. Marker *k*-mers with the count stored in *Q* equal to zero are omitted. Subsequently, the *k*-mers in all the selected reads from the *k*-mer counting step are enumerated. For each paired-end read, the set of its *k*-mers is enumerated, encoded into integers, and employed as indices to retrieve the corresponding cBKP loci stored in *B*. If two cBKPs from different species are retrieved through the same paired-end read, they are considered as HGT-derived cBKPs. The HGT-related segments are obtained by retrieving 500 bp sequences both upstream and downstream of all HGT-derived cBKPs from the reference genome.

#### Detection of precise HGT breakpoints

With reads aligned to the smaller HGT-related segments, as compared to the original reference database, we detect precise HGT breakpoints using a previous method (26) (Figure 1C). Here are the steps involved:

- Alignment: All sequencing reads are aligned to the HGT-related segments using BWA MEM (31). Reads with a mapping quality lower than 20 are discarded. The junction reads are extracted from the BAM file. A junction read refers to paired-end reads in which the two sides are mapped to two distinct genomes, enabling the inference of rough HGT breakpoint pairs.
- Clustering: We cluster rough HGT breakpoint pairs based on their positions in the associated genomes. Suppose there are *n* breakpoint pairs between species *A* and *B*. We use the genome of *A* as the x-axis and the genome of *B* as the y-axis. Each breakpoint pair has a position *x* on genome *A* and a position *y* on genome *B*. From the *n* breakpoint pairs, we obtain a two-dimensional variable (*X*, *Y*). The Euclidean distance ρ between two breakpoint pairs (*i* and *j*) is calculated as follows:
  (9) \begin{eqnarray*}
\rho = \sqrt{(x_i - x_j)^2 + (y_i - y_j)^2}
\end{eqnarray*}
  The breakpoint pairs are then clustered using the DBSCAN algorithm (32), with *epsilon* (neighborhood radius) set as 200 and *minPoints* (minimum number of points in a cluster) set as 1. Within each cluster, we obtain the lower and upper bounds of the breakpoint position.
- Realignment: To determine the precise position of the breakpoints, we utilize split reads. A split read can be divided into two parts, with one part being soft-clipped. We realign the soft-clipped part to the reference sequence using the Smith-Waterman algorithm (33), iterating from the lower to the upper bound of the possible breakpoint positions. The realignment process continues until the alignment score exceeds a cutoff threshold (default: 0.8), and the stop position represents the precise position of the breakpoint.

#### Detection of complete HGT events

We match the identified HGT breakpoint pairs to detect complete HGT events. An HGT event creates three breakpoints: two breakpoints on the donor genome, and one breakpoint on the recipient genome (Supplementary Figure S3). If two breakpoint pairs share a breakpoint and the other two distinct breakpoints are situated on the same genome, they might form an HGT event. The region between the two distinct breakpoints represents the transferred sequence, and the common breakpoint indicates the insertion site of the transferred sequence on the recipient genome. Such two breakpoint pairs fulfill the *link criteria*. However, even two independent breakpoint pairs might satisfy these link criteria, leading to ambiguity when inferring whether two breakpoint pairs originated from the same HGT event.

To address the ambiguity, we employ maximum-weighted matching from graph theory (Figure 1C). Given a sample, we model each HGT breakpoint pair as a vertex, and an edge connects two vertices if the two pairs satisfy the link criteria. The graph built is referred to as HGT breakpoint graph. The set of HGT events will correspond to a matching. However, the opposite statement may not hold true, as noise can exist within a graph. To further reduce the ambiguity, we impose the following constraints when we create an edge:

- The order between the breakpoint and the transferred sequence indicated by sequencing reads matches the order indicated by the estimated HGT event;
- The transfer direction (forward or reverse) of the transferred sequence is consistent between the two breakpoint pairs;
- The length of the estimated transferred sequence is at least 500 bp; and
- The estimated donor genome contains exactly two breakpoints related to the estimated insertion breakpoint. We collect all the breakpoints on the donor genome related to the estimated insertion breakpoint from the population, and cluster these breakpoints using DBSCAN algorithm (32) with parameters *epsilon* and *minPoints* set to 200 and 1, respectively. The number of resulting clusters should be exactly two.

The weight of an edge is defined as the average number of split reads of the two breakpoint pairs. We compute the maximum-weighted matching of the graph using the Networkx Python module (34). Each pair of matched nodes represents an HGT event.

### Large-scale HGT detection

To gain a comprehensive understanding of HGTs within the human gut microbiome, a total of 2098 shotgun metagenomic samples were collected from 17 cohorts, encompassing various phenotypes, including healthy, CRC, adenoma, T2D, IGT, acute diarrhoea (referred to as diarrhoea) and IBD (Table 1). LocalHGT was adopted to detect HGT breakpoints and events in these samples with default settings. The reference database utilized for HGT detection was the Unified Human Gastrointestinal Genomes (UHGG) v1 gut-specific representative genomes collection, as of December 2020 (35).

**Table 1.** Collection of gut microbiome data from different cohorts

Cohort	Country	Study	Accession number	No. of samples
ZellerG_2014	France	CRC	ERP005534	141
YuJ_2015	China	CRC	PRJEB10878	127
FengQ_2015	Austria	CRC	ERP008729	153
ThomasAM_2018a	Italy	CRC	SRP136711	80
ThomasAM_2018b	Italy	CRC	SRP136711	60
YachidaS_2019	Japan	CRC	DRA006684	78
WirbelJ_2018	Germany	CRC	PRJEB27928	128
VogtmannE_2016	USA	CRC	PRJEB12449	110
YangJ_2020	China	CRC	SRP128485	164
NielsenHB_2014	Europe	IBD	ERP002061	364
HallAB_2017	USA	IBD	PRJNA385949	147
KarlssonFH_2013	Europe	T2D	PRJEB1786	140
QinJ_2012	China	T2D	PRJNA422434	134
DavidLA_2015	Bangladesh	Acute diarrhoea	PRJEB9150	45
KieserS_2018	Bangladesh	Acute diarrhoea	PRJNA363003	27
cross-sectional cohort	China	Healthy	SRP366030	100
time-series cohort	China	Healthy	SRP366030	100
Total	-	-	-	2,098

The identified HGT breakpoints by LocalHGT exist as pairs, comprising a breakpoint on the donor genome and a corresponding breakpoint on the recipient genome. To ensure the reliability of the results, we filtered the HGT breakpoint pairs based on the supporting split reads. Specifically, breakpoint pairs with a ratio of split reads to total reads lower than 1e-7 were excluded from each sample. In line with previous studies (1,14), we adopted the concept of taxa pairs to analyze HGTs. A taxa pair signifies the occurrence of at least one HGT event between the genomes of two taxa. For example, if there is at least one HGT event between two species in a sample, that sample contains the respective species pair. Taxa pairs can be discerned at different taxonomic levels, including phylum pair, class pair, order pair, family pair, genus pair, and species pair.

### Taxonomic nomenclature

In this study, we ensured consistency in taxonomy names by utilizing the Genome Taxonomy Database (GTDB) R89 taxonomy system (36), which is employed in the UHGG v1 database. In the GTDB taxonomy, groups appended with alphabetical suffixes (e.g., Firmicutes_A) represent non-monophyletic groups in the GTDB reference phylogeny. Our analysis revealed that 94.1% (31,615/33,596) of the genomes containing both core organism names and alphabetical suffixes in the GTDB taxonomy are assigned corresponding core taxonomy names in the National Center for Biotechnology Information (NCBI) taxonomy. For example, 99.8% (4,698/4,706), 100% (245/245) and 100% (301/301) of the genomes in the phyla Firmicutes_A, Firmicutes_B, and Firmicutes_C are assigned to the phylum Firmicutes in the NCBI taxonomy, respectively. Furthermore, 98.78% (974/986) of the genomes labeled as *Escherichia coli_D* are assigned to the species *Escherichia coli* in the NCBI taxonomy. Therefore, to align with previous studies that did not utilize GTDB taxonomy, taxonomy names are considered the same if their core taxonomy name parts (without suffixes) match.

### Identification of MGEs associated with HGT events

To identify mobile genetic elements (MGEs) associated with HGT events, we conducted a search for known MGEs located adjacent to these events. We extracted the 5000 bp upstream and downstream flanking sequences surrounding the recipient breakpoint and the transferred sequence from the donor for each HGT event. These flanking sequences were aligned to the Intestinal Microbiome Mobile Element Database (ImmeDB) (37) using BLASTn (38) with default parameters. ImmeDB is a database that collects and annotates MGEs from gut microbiomes, including genomic islands, integrative mobilizable elements (IMEs), integrative conjugative elements (ICEs), transposons, and islets. Confident alignments were identified using an alignment *E*-value threshold below 1e-5. HGT events that had confident alignments to known MGEs in their vicinity were considered to be associated with those MGEs. Moreover, we applied the same approach to identify MGEs within the transferred sequence of each HGT event.

### Exploring mutational mechanisms for HGT events

To identify microhomology sequences at HGT breakpoint junctions, a comparison was made between the two sequences flanking the donor breakpoint and its associated recipient breakpoint. Specifically, for each breakpoint pair identified by the LocalHGT method, the sequence segment located within a 10 bp range from each breakpoint on the respective reference genomes was extracted. To identify the homology sequences, the Needleman-Wunsch algorithm was employed to align the two extracted sequences.

To assess whether an enrichment of microhomology existed in HGT breakpoint junctions, a comparison was made between the microhomology length of HGT breakpoint junctions and the expected background. The expected background was determined based on hypothetical breakpoint pairs created by randomly selecting two HGT breakpoints from the entire set of breakpoints. To perform this analysis, 10 000 HGT breakpoint pairs were randomly selected, and an additional 10 000 hypothetical breakpoint pairs were generated. The distributions of microhomology length between these two sets were then compared using the Wilcoxon rank-sum test. Furthermore, the ratio of microhomology sequences exceeding 5 bp was computed for each set. The distributions of microhomology lengths exceeding 5 bp between the two sets were compared using the Wilcoxon rank-sum test.

To determine the mutational mechanism underlying HGT events, an analysis of sequence patterns at breakpoints was conducted, following the methodology outlined in a previous study (39). The six possible mutational mechanisms are transposable element insertion (TEI), variable number of tandem repeats (VNTR), NHEJ, alt-EJ, nonallelic homologous recombination (NAHR), and fork stalling and template switching/microhomology-mediated break induced repair (FoSTeS/MMBIR) (39). To annotate transposable element regions within the UHGG database, Repeatmasker v2.0.1 was utilized (*http://www.repeatmasker.org*). Regions with descriptions containing ‘SINE’, ‘LINE’, ‘LTR’ and ‘transposon’ were classified as transposable elements. The tandem repeats were predicted using MISA microsatellite finder v2.1 (40).

### Quantification of HGT similarity between samples

We aimed to examine whether HGTs could serve as distinctive host fingerprints. To investigate this, a comparative analysis was conducted, examining the similarity of HGTs both between individuals (inter-personal) and within individuals over time (temporal intra-personal) within the time-series cohort. The cohort consisted of ten healthy individuals, each of whom was sampled at ten distinct time points (22). For each sample, its HGT similarity to nine other individuals (inter-personal) as well as nine temporal samples derived from the same individual (intra-personal) was computed.

Spearman’s correlation coefficient was employed as a measure of similarity for HGT breakpoint pairs. All HGT breakpoint pairs were gathered from the entire set of samples, and an array, denoted as *X*, was constructed for each sample. The variable *X*_*i*_ ∈ {0, 1} denoted whether the *i*-th HGT breakpoint pair was present in the sample. The Spearman’s correlation coefficient was computed for two samples by utilizing their respective arrays, with the calculation performed using the Python module scipy.stats.spearmanr. Subsequently, the correlation coefficient values obtained from inter-personal samples and intra-personal samples were compared using the Wilcoxon rank-sum test.

Moreover, the similarity of HGT events was quantified using the Jaccard similarity coefficient; that is, for HGT event sets *A* and *B* of two corresponding samples, the Jaccard similarity is


(10) \begin{eqnarray*}
J(A,B) = \frac{|A\ \cap \ B|}{|A\ \cup \ B|}.
\end{eqnarray*}

The Jaccard similarity coefficient of inter-personal samples and intra-personal samples was compared using the Wilcoxon rank-sum test.

### Functional annotation of HGT-related genes

To investigate the functional aspects of HGTs, a comprehensive functional annotation was carried out on HGT-related genes. The genes were classified based on their association with the ‘breakpoint’, the ‘transferred sequence’ and the ‘insertion site’. The ‘transferred sequence’ and ‘insertion site’ were obtained from identified HGT events. The ‘breakpoint’ category encompassed all identified HGT breakpoints, and genes containing these breakpoints within their intervals were collected. The same method was applied to collect genes related to ‘insertion site’. For ‘transferred sequence’, genes with over fifty percent of their length located on the transferred sequence were gathered. Subsequently, functional annotation was performed for each gene. The KO (KEGG Orthology) identifier, Clusters of Orthologous Genes (COG) category, and product information for each gene were extracted from the UHGG database. Based on the product description, the genes were classified into various categories such as carbohydrate-active enzymes (CAZYmes), phage, plasmid, transposon, antibiotic resistance, and other HGT mechanisms. The classification was accomplished using a text mining approach developed in previous studies (14,41).

Enrichment analyses for KEGG pathways, COG categories, and gene classifications were conducted utilizing Fisher’s exact test. Background genes for the breakpoint category were selected from non-breakpoint regions of the genomes involved in HGT breakpoints. The same approach was employed to select background genes for the transferred sequence and insertion sites. To determine the enrichment of a specific category (e.g. pathway, COG category, or classification) within a set of focused genes, the following methodology was employed. Assuming we have a set of focused genes, where *a* genes belong to the focused category and *b* genes do not belong to the focused category. In the background genes, *c* genes belong to the focused category, while *d* genes do not belong to the focused category. The probability of obtaining this particular set of values is as


(11) \begin{eqnarray*}
p = \frac{(a+b)!(c+d)!(a+c)!(b+d)!}{a!b!c!d!n!},
\end{eqnarray*}

where *n* = *a* + *b* + *c* + *d*. To control the false discovery rate, Bonferroni *P*-value correction was performed using the statsmodels.stats.multitest function in Python.

### Selection and functional analysis of disease-associated HGTs

To select disease-associated HGTs, differential HGT genus pairs were identified between disease-associated samples and control samples. A genus pair signifies the occurrence of at least one HGT event between the two genera. Due to the limited availability of species-level annotations in the UHGG database, our attention was directed towards genus pairs rather than species pairs. Genus pairs were collected from both sample groups, and the frequency of a genus pair within a specific group was determined by dividing the number of samples in which the genus pair was detected by the total number of samples in that group. To compare the frequencies of each genus pair between the two groups, Fisher’s exact test was conducted. Corrected *P*-values were obtained through Bonferroni method using the statsmodels.stats.multitest function in Python. Genus pairs with a corrected *P*-value of less than 0.05 were considered as differential HGT genus pairs between the two groups.

Functional analysis was performed on HGT genus pairs associated with diseases. All breakpoint pairs linked to differential HGT genus pairs were collected. Genes located within a 5000 bp vicinity of the collected breakpoints were extracted. KEGG pathway enrichment analysis was conducted for these genes, using the genes surrounding other breakpoints as the background. Furthermore, a previously established method was followed to screen for Short-Chain Fatty Acids (SCFAs)-related compounds based on the genes’ KO identifiers (42). The specific compounds of interest included pyruvate, acetyl-CoA, acetate, propionate, butyryl-CoA, succinate, lactate, and butyrate. The enrichment of these compounds was evaluated using Fisher’s exact test, and the resulting *P*-values were adjusted using the Bonferroni method. Additionally, a similar approach was adopted to investigate the enrichment of Shiga toxin-producing genes (*stxA* and *stxB*).

### Construction and validation of the disease classifier

To assess the potential of HGTs as disease biomarkers, differential HGT genus pairs (i.e., disease-associated HGTs) between disease and healthy groups were employed as predictive biomarkers for disease identification. The presence or absence of each genus pair was encoded as binary values (1 or 0) within the samples, representing the biomarker status for the classifiers. To mitigate bias arising from imbalanced data, under-sampling of the majority class was performed when dealing with two sample groups. A five-fold cross-validation approach was utilized to train and evaluate the classifier. The Random Forest binary classifier was chosen as the prediction model in this investigation. Implementation of the classifier was conducted using the scikit-learn Python module, with the parameter n_estimators=100. Furthermore, the classifier’s performance was assessed by incrementally increasing the number of biomarkers. For each biomarker count, ten rounds of cross-validation were performed, and the average AUC value was computed. Moreover, a separate Random Forest binary classifier was developed for each pair of diseases. These classifiers were constructed using the differential HGT genus pairs specific to each disease pair as biomarkers. To evaluate the performance of each classifier, ten iterations of five-fold cross-validation were executed.

Furthermore, the integration of HGTs and microbial-abundance biomarkers was evaluated for its utility in the CRC cohorts. We collected 16 microbial-abundance CRC biomarkers from previous research (43), as well as 16 of the most differential HGT genus pairs between CRC and control. The genera involved in these genus pairs were also considered as abundance biomarkers. The microbial abundances were obtained from the curatedMetagenomicData v3.0.4 database (44). To refine the values of the abundance biomarkers, graph signal processing techniques were applied. The Pearson correlation coefficient between every two taxa was computed by comparing their abundances across all the samples. Coefficients below 0.4 were set to 0, resulting in a refined correlation matrix denoted as *C*. The refined abundances were obtained using the equation $y = (\zeta C + I) \hat{y}$, where $\hat{y}$ indicates the raw abundance array, *I* represents the identity matrix, and ζ was set to 0.048. To predict CRC, the existence status of the HGT genus pairs and the refined microbial-abundance values were concatenated into a single vector.

To evaluate the classifier’s robustness against batch effects, a leave-one-dataset-out (LODO) approach was employed. Out of the nine collected CRC cohorts, eight were used for the LODO analysis, and the remaining one was utilized as independent validation data. Among the eight CRC cohorts in LODO analysis, one cohort was selected at a time as the testing dataset, while the remaining seven cohorts served as the training datasets. The classifier was implemented with specific parameter settings, including n_estimators = 1000, criterion=entropy, min_samples_leaf = 5, max_features = 2. For each testing cohort, the AUC was computed, and the average AUC was calculated in a weighted manner, taking into account the sample size of each testing cohort. To further validate the classifier, it was trained using all eight CRC cohorts and subsequently tested on the independent CRC cohort as well as a cohort related to T2D.

### Construction and analysis of populational HGT networks

To explore the relationship between HGT networks and human diseases, two types of undirected HGT networks were constructed: populational networks and individual networks. The populational HGT network was built based on all samples within a specific group, while the individual HGT network was constructed for each individual sample. In the populational HGT network, taxa were represented as nodes, and edges represented the frequency of HGT events occurring between two taxa within the group. Subsequently, the identification of important taxa within the populational HGT network was performed. A taxon frequently involved with HGTs or transferring sequences with diverse taxa was considered significant. Furthermore, the importance of a taxon could be inferred from its connections to other important taxa within the network. To assess the significance of taxa in the network, the PageRank (PR) algorithm was applied. The PR score was utilized to quantify the importance of each taxon.

### Construction and analysis of individual HGT networks

Individual HGT networks were created for each sample using a methodology similar to prior research (45). In these networks, taxa were represented as nodes, while edges indicated the presence of at least one shared HGT event between two taxa. The construction of HGT networks involved annotating taxa at the phylum level. To ensure a fair comparison of networks, an equivalent number of edges was retained in each network. The count of supporting reads for all HGT breakpoint pairs between the two phyla was tallied. Edges with the highest count of supporting split reads were preserved in each network. An edge threshold of ten was set, and networks containing fewer edges than the threshold were discarded.

To quantify the difference between HGT networks, several topological properties of the HGT network were computed. These properties include density, transitivity, degree assortativity, and algebraic connectivity. Density measures the ratio of edges in the network to the maximum possible number of edges (46). Given a network *G* = (*V*, *E*), the density can be calculated as


(12) \begin{eqnarray*}
Density = \frac{|E|}{\binom{|V|}{2}}.
\end{eqnarray*}

Transitivity measures the probability that the adjacent vertices of a vertex are interconnected. It is determined by the ratio of observed triangles to the maximum number of triangles possible in the network. Degree assortativity refers to the tendency for nodes in a network to be connected to nodes with similar degrees. We quantified the assortativity by the Pearson correlation coefficient of the degree–degree correlation. Algebraic connectivity, which corresponds to the second-smallest eigenvalue of the Laplacian matrix of the network, was also considered as a topological property. The topology properties were calculated using the Python module NetworkX. Subsequently, a comparison of HGT network properties between two groups was conducted using the Wilcoxon rank-sum test implemented in the scipy.stats.ranksums function of Python. To mitigate the issue of false discovery, Benjamini-Hochberg *P*-value corrections were applied using the statsmodels.stats.multitest Python module.

Furthermore, the investigation focused on determining whether the HGT network exhibits a scale-free property. Previous research has provided evidence that HGT networks, where reference genomes serve as nodes (at approximately the species level), exhibit a scale-free property (45). Our research sought to ascertain whether the HGT network constructed in our study also demonstrates a scale-free nature. This was accomplished by examining the degree distribution and assessing its fit to a power-law distribution (45). To assess the adequacy of fitting the degree distribution to multiple assumed distributions including power-law, exponential, lognormal, and Weibull distributions, the log-likelihood ratio test was employed. The presence of a scale-free property in the network was established when the degree distribution demonstrated a better fit to the power-law distribution compared to the alternative assumed distributions.

## Results

### LocalHGT enables reliable and swift detection of complete HGT events

**Figure 2.**
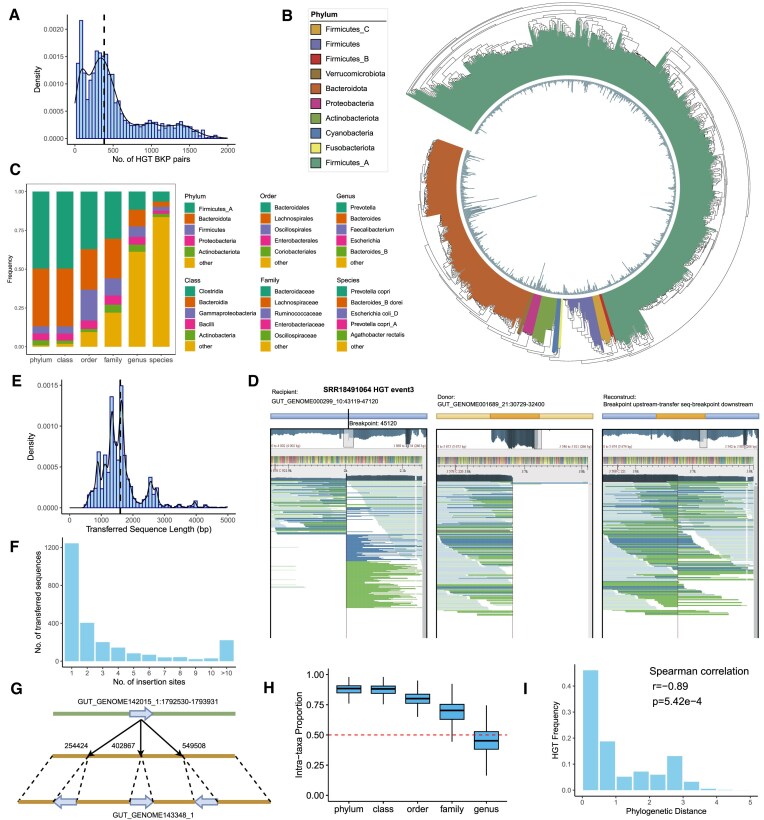
Characterization of HGTs within the human gut microbiome. (**A**) Distribution of the number of HGT breakpoint pairs in different samples. The black dashed line indicates the median value. (**B**) Phylogenetic tree of the 842 genomes involved in HGT in no less than 10% of the samples. Colors indicate the corresponding phylum of each genome. The height of the inside bar represents the average HGT frequency among samples. (**C**) HGT frequency of different taxa at the phylum, class, order, family, genus, and species levels. Only the top five taxonomic units are shown for each taxonomic level, while the remaining taxa are labeled as ‘other’. (**D**) Schematic representation of the reconstructed local genome using the inferred HGT event, reads alignment visualized with UGENE (89). (**E**) Distribution of the length of transferred sequences in HGT events. The black dashed line indicates the median length. (**F**) Distribution of transferred sequences with various numbers of target insertion sites. (**G**) Illustration of an example showing a transferred sequence has three target insertion sites within the recipient genome. The blue arrow represents the transferred sequence. The transferred sequence is inserted into the recipient genome twice in a reverse complement manner. (**H**) Intra-taxa HGT proportion at different taxomomic levels. The red line indicates a proportion of 50%. (**I**) HGT frequency of the genome pairs at different phylogenetic distance levels. The x-axis indicates the phylogenetic distance bins and the y-axis shows the HGT frequency.

**Figure 3.**
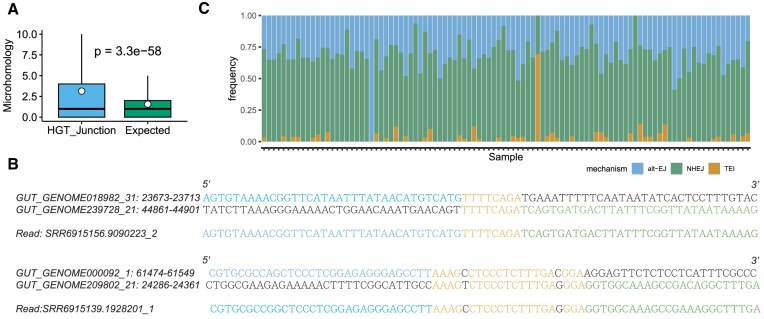
Inference for HGT mechanisms. (**A**) Comparison of microhomology length of HGT breakpoint pair junctions (HGT-Junction) and expected background (expected). (**B**) Example of microhomologous sequences at two HGT breakpoint junctions. The top two sequences represent the original reference genomes, and the bottom sequence shows the fused sequence observed from the sequencing read. Orange indicates the microhomologous sequences. The exact breakpoint position cannot be identified in both breakpoint junctions because the breakpoint is located inside the microhomologous sequence. Both breakpoint junctions are detected in >100 samples. (**C**) Frequencies of different HGT deletion mechanisms. The vertical bar displays the frequencies of mechanisms for each sample. Each color represents one type of mechanism.

LocalHGT is for the reliable and efficient detection of complete HGT events from shotgun metagenomic data. It accurately captures the transferred sequence and identifies the associated deletion and insertion sites in the donor and recipient genomes, respectively. LocalHGT consists of three main components (Methods): (1) a novel *k*-mer encoding algorithm that allows fuzzy *k*-mer matching to tolerate substitutions (Figure 1A), (2) a procedure to extract HGT-related segments, i.e., reference segments potentially containing HGT breakpoints, from the reference database using fuzzy *k*-mer matching (Figure 1B), and (3) a pipeline that deduces precise HGT breakpoint loci by aligning reads to the HGT-related segments and infers complete HGT events by matching HGT breakpoint pairs (Figure 1C).

The workflow of LocalHGT, as depicted in Supplementary Figure S1, can be summarized as follows. Initially, given a sample, LocalHGT conducts *k*-mer counting in the sequencing reads, followed by enumerating *k*-mers along the genome in the reference database to map the count of each *k*-mer onto the reference (Figure 1B). Candidate breakpoints (cBKPs) are selected based on genomic loci exhibiting significant changes in *k*-mer count along the reference. The *k*-mers at these cBKP loci are collected as markers. Subsequently, a re-enumeration of *k*-mers in the sequencing reads is performed. If the marker *k*-mers obtained from two distinct cBKPs are identified within the same paired-end read, and these two cBKPs correspond to different species, they are classified as HGT-derived cBKPs. The reference segments surrounding these HGT-derived cBKPs are extracted to form a collection of HGT-related segments. To map the sequencing reads to these segments, BWA MEM is employed, and the precise positions of HGT breakpoints are inferred using junction reads (Figure 1C). Finally, the HGT breakpoint graph is introduced, where each HGT breakpoint pair is represented as a node, and the presence of an edge indicates the potential formation of an HGT event (Figure 1C). Complete HGT events are identified by performing maximum weighted matching on this graph.

We have extensively validated LocalHGT and confirmed its capacity to achieve precise HGT detection with exceptional computational efficiency (Supplementary Note S2 and Supplementary Figures S4–S9). The benchmark experiment results can be summarized as follows:

- LocalHGT accurately detects complete HGT events with a remarkable accuracy of 99.4% (4,748/4,775) in 200 gut metagenomic samples. The accuracy was assessed with matched Nanopore long-read sequencing data. Furthermore, the F1 score of LocalHGT in detecting HGT events is 0.99 in 100 simulated samples (Supplementary Figure S4). Additionally, LocalHGT exhibits a higher F1 score for HGT breakpoint detection compared to LEMON (Supplementary Figure S5).
- LocalHGT demonstrates exceptional efficiency. In the Critical Assessment of Metagenome Interpretation (CAMI) datasets, LocalHGT significantly outperforms the traditional alignment-based tool LEMON in terms of resource utilization (Supplementary Figure S6). On average, LocalHGT required 73.8% less wall-clock time, 82.7% less CPU time, and 2.7% less memory. LocalHGT’s efficiency becomes increasingly evident as the sequence output amounts grow larger (Supplementary Figure S7). Moreover, it has been determined that a sequencing depth of 30x is the recommended requirement for the genome targeted by LocalHGT (Supplementary Figure S8). This information can assist users in estimating the suitable amount of sequence output necessary for the HGT detection conducted by LocalHGT (Discussion).
- LocalHGT’s advantage becomes more pronounced when handling large reference databases. With a reference database size of 45.4G, LocalHGT consumed significantly lower memory (22.7G) compared to LEMON (79.0G).
- LocalHGT demonstrates robustness across a wide range of factors, including sequencing depth, insert size, read length, the divergence distance between the local genome and the reference, as well as the presence/absence of the donor bacteria (Supplementary Figure S5 and S9).

### Transferred sequences of HGTs can have multiple target insertion sites

Using LocalHGT, we conducted a comprehensive analysis of HGTs in the gut microbiome, focusing on 2098 samples (Methods, Table 1, Supplementary Table S1 and Supplementary Figure S10). These samples were collected from diverse populations across three continents and over eight countries, encompassing various microbiome-associated diseases such as CRC, IBD, T2D and acute diarrhoea. This broad range of samples allowed us to gain a comprehensive understanding of HGTs. Our analysis of HGTs involved examining HGT breakpoints, complete HGT events, the phylogenetic structure of genomes involved in HGT, and the frequency of HGT among different taxonomic groups. Based on the HGT events identified in these samples, we identified two patterns associated with HGT: first, a transferred sequence can be inserted into multiple sites within recipient genomes, and second, the frequency of HGT is negatively correlated with the phylogenetic distance between the genomes involved. Additionally, while inter- and intra-phylum HGT events are closely linked to mobile genetic elements (MGEs), the involvement of MGEs differs between these events.

Our analysis confirmed the widespread occurrence of HGT events within the gut microbiome, while also revealing the phylogenetic relationships among the genomes involved in HGT and identifying the taxa most frequently associated with HGTs. On average, each sample contained 559 HGT breakpoint pairs discovered by LocalHGT (median 383, range 2 to 10 018, Figure 2A). Since each HGT event contributes to two HGT breakpoint pairs, it is likely that each sample harbored an average of 280 HGT events. These findings align with previous studies (14,15) that also reported extensive HGT events in the gut microbiome. We then constructed a phylogenetic tree to represent the genomes involved in HGT. We collected 842 genomes that were involved in HGT in at least 10% of the samples. The majority of these genomes belonged to the phylum Firmicutes_A, followed by Bacteroidota (Figure 2B). This is consistent with the overall gut microbiota composition, where Firmicutes and Bacteroidota (referred to as Bacteroidetes in the cited paper) collectively represent 90% of the microbiota (47). In the GTDB taxonomy used in this study, groups that are appended with alphabetical suffixes indicate non-monophyletic groups in the GTDB reference phylogeny (36). To align with prior research, taxonomy names are deemed identical when their core taxonomy name components (excluding suffixes) correspond (see Methods). Moreover, to identify the taxa most frequently associated with HGTs in the gut microbiome, we introduced the concept of HGT frequency. The HGT frequency of a taxon refers to the number of HGT breakpoints involved in its genome divided by the total number of breakpoints in each sample. We calculated the average HGT frequency across all samples. Figure 2C presents the top five taxa with the highest HGT frequency at each taxonomic level. Among taxa at the same level, phylum Firmicutes_A (49.7%), class Clostridia (49.7%), order Bacteroidales (37.2%), family *Bacteroidaceae* (30.4%), genus *Prevotella* (11.7%) and species *Prevotella copri* (6.6%) exhibited the highest HGT frequency. Notably, the extensive HGTs observed in the order Bacteroidales and the phylum Firmicutes_A (Firmicutes in the cited study) align with previous studies (22,48).

The HGT events identified by LocalHGT indicate that a transferred sequence can be inserted into multiple sites within recipient genomes. In total, LocalHGT detected 19 343 complete HGT events across 2098 samples. Figure 2D showcases the breakpoint junction and aligned reads of an HGT event in sample SRR18491064. Among these HGT events, the median length of the transferred sequence is 1600 bp (Figure 2E). These events involved 2495 distinct transferred sequences. Interestingly, a transferred sequence can target multiple insertion sites. When examining the distinct target insertion sites for each transferred sequence across all samples, 50.2% (1252/2495) of the transferred sequences had more than one target insertion site (Figure 2F). Moreover, even within an individual, a transferred sequence can possess multiple insertion sites. By counting the insertion sites for each transferred sequence within each individual, 26.7% (665/2495) of the transferred sequences displayed multiple insertion sites within an individual. Additionally, 20.8% (519/2495) of the transferred sequences exhibited multiple insertion sites within the same recipient genome within an individual. Figure 2G illustrates an example where a transferred sequence has multiple insertion sites within the same recipient genome in sample SRR18491280.

Moreover, it appears that the multiple transfer events involving a transferred sequence within a single individual tend to arise as novel occurrences within an individual. If an HGT event newly occurs within an individual, it is unlikely to be shared by multiple individuals. Among the 1252 distinct transferred sequences capable of targeting multiple insertion sites, we identified 10 058 related HGT events. Interestingly, a significant majority of these events (82.5% or 8301 out of 10 058) were observed in only one individual. Hence, the presence of multiple transfer events of a transferred sequence within a single individual is more likely to newly occur in that specific individual.

Furthermore, a significant proportion of HGT events occurred within the same taxonomic group (intra-taxa), and the HGT frequency negatively correlates with the phylogenetic distance of the involved genomes. To clarify, we consider an HGT event as intra-taxa if the two genomes involved belong to the same taxon. Across all samples, the average proportions of intra-taxa HGTs were 84.7%, 84.4%, 77.2%, 67.2% and 45.3% at the phylum, class, order, family, and genus levels, respectively, as inferred from HGT breakpoint pairs (Figure 2H). The considerable proportion of intra-taxa HGTs observed at varying levels indicates that numerous HGT events take place among genomes that are taxonomically closely related. Additionally, we calculated the HGT frequency and the phylogenetic distance for each pair of genomes (Figure 2I). The HGT frequency represents the number of HGT breakpoint pairs between the two genomes divided by the total number of HGT breakpoint pairs in a sample. We computed the average HGT frequency for each pair of genomes across all samples. The average HGT frequency has a negative correlation with the phylogenetic distance (Spearman’s correlation, *r*= –0.89, *P*-value = 5.4e-4). This indicates that HGT events are more likely to occur between genomes that are phylogenetically closer, supporting previous findings (49).

Additionally, MGEs are vital in facilitating HGT events and MGEs might exhibit distinct roles in driving inter- and intra-phylum HGT events. Out of the complete HGT events identified by LocalHGT, 10.3% (1986/19 343) were found to be inter-phylum. MGEs were found to be abundant in the transferred sequences of both inter- and intra-phylum HGT events, with the categories of MGEs varying between these two types of HGTs. In total, 22.9% (454/1986) and 29.0% (5039/17 357) of the transferred sequences contained MGEs in inter- and intra-phylum HGT events, respectively. Notably, transposons were significantly more abundant in the transferred sequence of inter-phylum HGT events than intra-phylum HGT events (77.3% versus 46.8%, Fisher’s exact test, *P*-value = 1.7e-33, Supplementary Figure S11). Remarkably, the transposon NZ_GG703857.1_43343_44503_Transposons305, identified from *Prevotella copri* DSM 18205 in the ImmeDB database (37), constituted 59.0% (268/454) of the MGEs within the transferred sequences involved in inter-phylum HGT events (see detail in Supplementary Note S3 and Supplementary Figure S11).

### Microhomology is enriched in HGT breakpoint junctions

This study revealed the enrichment of microhomology at the HGT breakpoint junctions within the gut microbiome. Additionally, it suggested that NHEJ and alt-EJ are the primary mutational mechanisms responsible for driving the formation of HGT events.

HGT breakpoint junctions harbored a significantly higher level of microhomology than expected. Existing research has found microhomology, which refers to short DNA sequence homology, is enriched at breakpoint junctions of structural variants in human genomes (50,51). We are interested in investigating whether there is an enrichment of microhomology in the breakpoint junctions of HGTs within the gut microbiome. To ascertain if an enrichment of microhomology occurred in HGT breakpoint junctions, a comparison between the microhomology distribution of HGT breakpoint junctions and the expected background took place (51). The background was based on hypothetical breakpoint pairs constructed by randomly choosing two HGT breakpoints among all the breakpoints. The HGT breakpoint junctions exhibited a higher level of microhomology than expected by chance, with an average of 3.1 bp versus 1.6 bp (a 1.9-fold increase, Wilcoxon rank-sum test, *P*-value = 3.3e-58, Figure 3A). Especially, the enrichment of microhomology exceeding 5 bp was significantly more pronounced in HGT breakpoint junctions compared to the expected background (Wilcoxon rank-sum test, *P*-value = 8.3e-124). The ratio of microhomology exceeding 5 bp was 16.6% in HGT breakpoint junctions, whereas it was 6.0% in the expected background. Figure 3B illustrates the microhomologous sequences at two HGT breakpoint junctions. The enrichment of microhomology at HGT breakpoint junctions suggested microhomology-mediated mechanisms play an important role in the HGT event formation (52), which has inspired us to delve deeper into investigating the mutational mechanisms underlying HGT events.

Complete HGT events identified by LocalHGT revealed that NHEJ and alt-EJ emerged as primary mutational mechanisms for HGT event formation. To investigate the mutational mechanisms of HGT events, we obtained 2320 verified HGT events from the cross-sectional cohort (22), and assigned mechanisms for these HGT events. Previous research has reported a method to assign mechanisms for deletion and insertion in structural variations (39). Following this approach, we divided each HGT event into a deletion and an insertion, enabling us to independently predict the mechanisms for deletion and insertion (Methods). Among all the HGT events, NHEJ (66.7%) and alt-EJ (30.6%) were the dominant mechanisms for deletion formation, with TEI accounting for the remaining cases (2.7%). Only five insertions were assigned mechanisms, one as VNTR and four as TEI. This contrasts with the somatic deletion mechanisms in human cancer genomes, where alt-EJ is the most dominant mechanism (41%), followed by NHEJ (39%) (39). The finding also differs from the mechanisms observed in non-tumor human genomes, where TEI is the most dominant mechanism (39). The ratio of NHEJ increases significantly in bacterial HGT events. VNTR, NAHR, and FoSTeS/MMBIR mechanisms were not observed, suggesting that these mechanisms may not be involved in HGT event formation in bacteria. We did not observe any differences in the frequency of mechanisms across various bacterial lineages. The frequency of NHEJ and alt-EJ ranged from 0% to 100% in different samples, while TEI had a range of 0% to 69.2% (Figure 3C).

### HGTs are personalized and associated with microbial adaptation

HGTs within the gut microbiome exhibit two distinct properties. Firstly, they can serve as personalized signatures of the host, as revealed by analyses conducted on a time-series cohort. Secondly, HGT events are closely associated with microbial adaptation, as indicated by the enrichment of genes related to HGTs in defense mechanisms and secretion functions.

**Figure 4.**
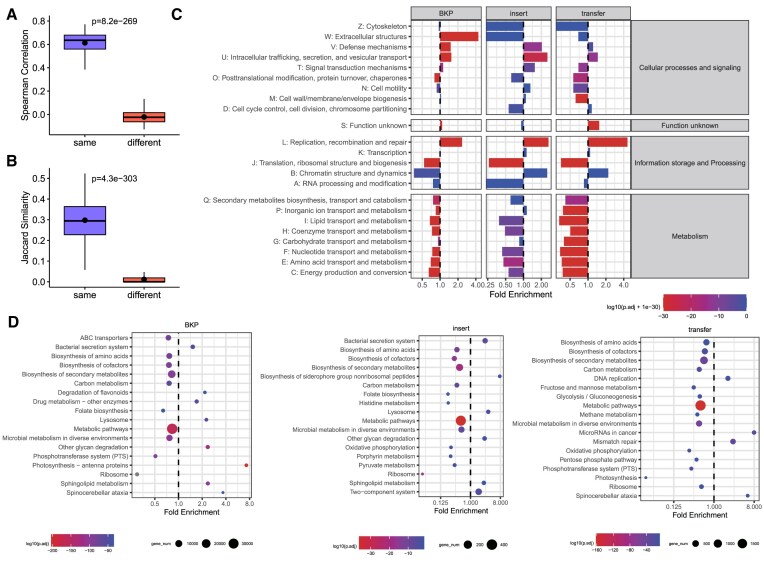
HGTs are person-specific and function-informative. (A, B) Comparisons of HGT breakpoint similarity (**A**) and HGT event similarity (**B**) between intra-personal (same) and inter-personal (different) samples. (**C**) Differential COG functional categories between background genes and genes surrounding HGT ‘breakpoint’ (BKP), ‘insertion site’ (insert), and ‘transferred sequence’ (transfer). The right boxes indicate the profiles of the COG categories. (**D**) Differential KEGG pathways between background genes and genes surrounding HGT ‘breakpoint’ (BKP), ‘insertion site’ (insert), and ‘transferred sequence’ (transfer). The pathways to the right of the dashed line indicate enriched pathways, and the left represents depleted pathways. The color indicates the *P*-value of the enrichment, and the bubble size represents the number of supporting genes. Only the top 18 most differential pathways are displayed.

Both HGT breakpoints and HGT events detected by LocalHGT exhibit time-stable and person-specific characteristics. Previous studies have discovered that SNP haplotypes and SVs of gut microbiome exhibit temporal stability and inter-personal variability and can thus serve as host fingerprints (22,53). We attempted to investigate whether HGTs can also function as host fingerprints by comparing inter-personal and temporal intra-personal HGT similarity in the time-series cohort. The time-series cohort comprises ten healthy individuals, where each individual was separately sampled from ten time points (22). The HGT breakpoint similarity between samples was quantified by Spearman’s correlation coefficient. Notably, the Spearman’s correlation coefficient of intra-personal samples is significantly higher than that of inter-personal samples (Mann–Whitney *U* test, *P*-value = 8.2e-269). The median correlation of intra-personal samples is 0.64 whereas that of inter-personal samples is –0.02 (Figure 4A). Moreover, the HGT event similarity between samples was measured by the Jaccard similarity coefficient (Figure 4B). The Jaccard similarity coefficient of intra-personal samples (median value: 0.29) is significantly higher than that of inter-personal samples (median value: 0) (Mann–Whitney *U* test, *P*-value = 4.3e-303). These results suggested that, similar to SNP haplotypes and SVs, HGT events are also highly person-specific and time-stable microbiome signatures.

COG category and KEGG pathway enrichment analyses suggested HGTs are associated with microbial adaptation. We systematically characterized the function of HGTs by searching for the genes surrounding ‘breakpoint’, ‘insertion site’, and ‘transferred sequence’ from all 2098 samples (Methods). ‘Breakpoint’ refers to all the detected HGT breakpoints, while the ‘insertion site’ and ‘transferred sequence’ were obtained from the inferred complete HGT events. Genes located in non-breakpoint regions of HGT-involved genomes were considered as background genes for ‘breakpoint’ analysis, and a similar approach was used to select background genes for ‘insertion site’ and ‘transferred sequence’. According to the COG and KEGG analyses, HGT-related genes exhibited a depletion of housekeeping functions but an enrichment of microbial adaptation-related functions. The COG category analyses indicated that the ‘breakpoint’, ‘insertion site’, and ‘transferred sequence’ were enriched in the categories *V: Defense mechanisms* and *U: Intracellular trafficking, secretion, and vesicular transport*, while experiencing a depletion of housekeeping functions (Figure 4C). In the KEGG analyses, the ‘breakpoint’, ‘insertion site’, and ‘transferred sequence’ simultaneously showed an enrichment of the bacterial secretion system and a depletion of housekeeping functions such as metabolic pathways and biosynthesis of secondary metabolites (Figures 4D, Table S2). Moreover, there was a depletion of CAZYmes-related genes in the ‘transferred sequence’ (Supplementary Figure S12). Conversely, genes associated with transposons showed enrichment in the ‘breakpoint’, ‘insertion site’, and ‘transferred sequence’. The transfer of transposons among prokaryotes represents a significant mechanism for generating genetic diversity and playing a pivotal role in prokaryote evolution (54). These functional findings consistently suggested the association between HGTs and microbial adaptation, which is in accordance with previous studies (1,2).

### Disease-associated HGTs are enriched in important KEGG pathways

Through the association analyses between HGTs and diseases, we identified the butyrate metabolism pathway is enriched in HGTs associated with CRC, and the shigellosis pathway is enriched in HGTs associated with acute diarrhea, indicating potential contributions of HGTs to these diseases via these pathways. To identify disease-associated HGTs, we employed the concept of the genus pair, which signifies the presence of at least one HGT event between two genera. After discarding the sample with multiple diseases, we classified the samples into different groups, including healthy controls (994 samples), CRC (415 samples), adenoma (51 samples), IGT (49 samples), T2D (116 samples), acute diarrhoea (54 samples), and IBD (217 samples). We calculated the frequency of each genus pair in a group by dividing the number of samples where the genus pair was present by the total number of samples in that group (Methods). Subsequently, we compared the frequency of HGT genus pairs between each disease group and the control group. We identified the differential HGT genus pairs using Fisher’s exact test, with a Bonferroni-corrected *P*-value threshold of <0.05.

**Figure 5.**
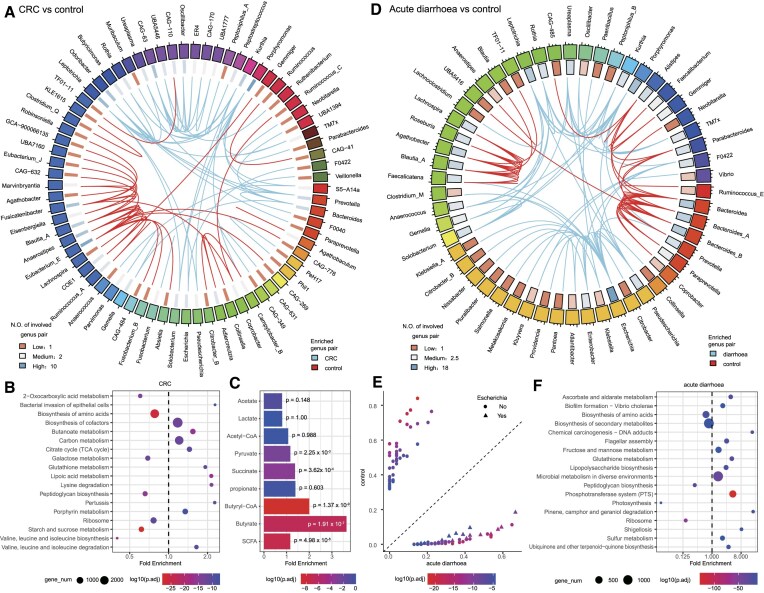
HGTs are enriched in important KEGG pathways of human diseases. (**A**) Illustration of the differential genus pairs between CRC and control. Each cell in the first track represents a genus, and genera of the same family are marked with the same color. The gradient color in the second track represents the number of involved differential genus pairs of each genus. The inner line indicates the genus pairs. The sky-blue line means the genus pair is enriched in CRC and red line means the genus pair is enriched in controls. (**B**) Differential KEGG pathways in genes surrounding HGT breakpoints of CRC-enriched genus pairs. Only the top 18 differential pathways are displayed. (**C**) Enrichment of SCFA-related compounds in genes surrounding HGT breakpoints of CRC-enriched genus pairs. The SCFA category in the y-axis means taking all compounds as a whole. (**D**) Illustration of the differential genus pairs between acute diarrhoea and healthy controls. (**E**) Frequency of differential genus pair in acute diarrhoea and healthy controls. (**F**) Differential KEGG pathways in genes surrounding HGT breakpoints of *Escherichia*-involved differential genus pairs.

CRC-associated HGTs are associated with multiple bacteria that have previously been identified as related to CRC. Between CRC and controls, we identified 85 differential HGT genus pairs in total, in which 52 genus pairs were enriched in CRC and 33 were depleted in CRC (Figure 5A). The most significantly differential genus pair was intra-*Fusobacterium* (Fisher’s exact test, Bonferroni-corrected *P*-value=2.4e-18). 72 genera were involved in these differential genus pairs, and among them, 18 genera belonged to the family *Lachnospiraceae*. All 27 genus pairs involved with this family were depleted in CRC. The genus *Porphyromonas* was involved in ten differential genus pairs and all of them were enriched in CRC. All of the nine genus pairs involved with the genus *Eubacterium_E* were CRC-depleted. Notably, the family *Lachnospiraceae*, and the genera *Fusobacterium*, *Porphyromonas* and *Eubacterium* have been previously found to be associated with CRC (19,55–57).

The functional analysis revealed that HGTs could potentially disrupt the production of Short-Chain Fatty Acids (SCFAs) compounds, thereby promoting the progression of CRC. Assume HGT breakpoints belong to the CRC-enriched genus pairs as CRC-enriched HGT breakpoints. We then performed KEGG pathway enrichment analyses on the genes surrounding CRC-enriched HGT breakpoints, with the background genes as those surrounding all other HGT breakpoints, resulting in 45 differential KEGG pathways (Figure 5B, Supplementary Table S3). Lipoic acid metabolism (Fisher’s exact test, Bonferroni-corrected *P*-value = 1.3e-17) was the most significantly enriched pathway, followed by the butanoate (i.e., butyrate) metabolism pathway (Fisher’s exact test, Bonferroni-corrected *P*-value = 3.8e-17). It is well known that the reduction of butyrate production contributes to the structural imbalance of gut microbiota in CRC patients (47). The enrichment of the butyrate pathway in CRC-enriched HGTs suggested that HGT events might harm the butyrate production in CRC patients. Butyrate is a member of SCFAs, and several SCFA-related compounds have been demonstrated to reduce CRC risk (58). Furthermore, we investigated the distribution of each SCFA-related compound on CRC-enriched HGTs by examining the KOs related to each SCFA-related compound (Methods). The butyrate was the most enriched SCFA-related compound, followed by butyryl-CoA, succinate, and pyruvate (Figure 5C). The functional analysis showed that HGTs might damage the production of various SCFA-related compounds in the gut microbiome of CRC patients, potentially contributing to the progression of CRC.

Moreover, the association analysis uncovered a potential contribution of HGTs to acute diarrhea by means of virulence factors associated with *Shigella*. We identified 106 HGT genus pairs enriched and 101 depleted in acute diarrhoe compared to controls (Figure 5D). The most enriched genus pair was between the genera *Kurthia* and *Leptotrichia* (Fisher’s exact test, Bonferroni-corrected *P*-value = 3.2e-18), of which the frequency was 53.7% and 4.3% in acute diarrhoe and controls, respectively. The genus *Escherichia* was most involved in these differential genus pairs, being involved in 18 of them, and all the 18 genus pairs were enriched in acute diarrhoe (Figure 5E). Notably, the vital pathogenic role of *Escherichia* members in acute diarrhoe has been found by previous studies (59,60). Furthermore, we investigated the possible functional link between *Escherichia*-related HGTs and acute diarrhoe. We performed KEGG pathway enrichment analysis on genes surrounding the HGT breakpoints belong to the 18 *Escherichia*-involved differential genus pairs, with the genes surrounding all other HGT breakpoints as background. Totally, 74 differential KEGG pathways were identified (Figure 5F, Supplementary Table S4). Interestingly, the pathway shigellosis (entry: hsa05131), which denotes the mechanism through which *Shigella* bacteria invade human intestinal cells, displayed a significant enrichment in acute diarrhea-associated HGTs (Fisher’s exact test, Bonferroni-corrected *P*-value = 5.9e-13). *Shigella* infection has been well known to cause severe diarrhea (61). Genetically, *E. coli* and *Shigella* species are considered to be the same species (62). It is reported that *E. coli* strains become diarrheagenic by acquiring Shiga toxin genes through HGT events (6,7). Additionally, we discovered a significant enrichment of Shiga toxin-producing genes (*stxA* and *stxB*) in *Escherichia*-related and diarrhoe-enriched HGTs (Fisher’s exact test, *P*-value=8.1e-8). Therefore, we hypothesize that in addition to *E. coli*, other *Escherichia* species could acquire Shiga toxin genes through HGT and potentially contribute to the development of diarrhea. Furthermore, we exhibited the functional link between HGTs and IBD as well as IGT (Supplementary Table S4 and Supplementary Figures S13–S14). The findings demonstrate the association of HGTs with various human diseases and their potential to provide mechanistic insights into the understanding of these diseases.

### HGTs can be promising biomarkers for diseases

**Figure 6.**
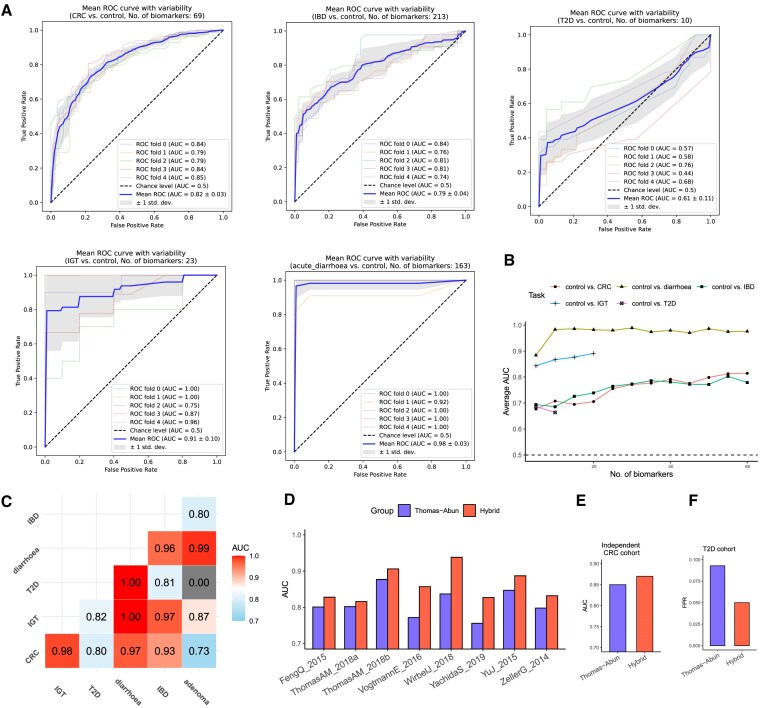
Evaluation of disease prediction models using HGTs as biomarkers. (**A**) ROC curves of the models for predicting CRC, IBD, T2D, IGT, and acute diarrhoea. (**B**) The AUC with the increasing number of differential HGT genus pairs for each classifier. (**C**) Average AUC of the models used to differentiate between different diseases. (**D**) Performance comparison of the classifier using only abundance biomarkers (‘Thomas-Abun’) versus incorporating both abundance and HGT biomarkers (‘Hybrid’) in the LODO analysis. (**E**) The validation of the classifier in an external CRC cohort. (**F**) The validation of the classifier in an independent T2D cohort. The false positive rate (FPR) represents the proportion of the samples falsely predicted as CRC out of all the samples in the T2D cohort.

HGTs within the gut microbiome demonstrated the capacity as biomarkers to predict various human diseases. Differential HGT genus pairs between the disease and controls were utilized as biomarkers to predict each specific disease. The diseases include CRC, IBD, T2D, IGT and diarrhoea. Adenoma was excluded as it had no differential HGT genus pair with controls. Random Forest binary classifiers were constructed for each disease (Methods). The classifiers were evaluated using five-fold cross-validation, with the majority class balanced through undersampling. Following the balancing procedure, 69, 213, 10, 23 and 163 HGT biomarkers were identified for CRC, IBD, T2D, IGT and diarrhea, respectively. The biomarker values were determined based on the presence/absence of the differential HGT genus pair in the sample. The average AUC for predicting CRC, IBD, T2D, IGT, and diarrhoea were 0.82, 0.79, 0.61, 0.91 and 0.98, respectively (Figure 6A). These results indicated that HGT could serve as promising biomarkers to predict CRC, IBD, IGT, and diarrhoea, and it performed relatively poorly in predicting T2D. The impact of the number of biomarkers on classifier performance was also investigated. With the number of biomarkers increasing, the average AUC increased for predicting most of the diseases except for T2D (Figure 6B). Furthermore, the ability of HGTs to differentiate between various diseases was explored. Random Forest binary classifiers were constructed for each pair of diseases using differential HGT genus pairs as biomarkers. Ten iterations of five-fold cross-validation were performed, and the average AUC was calculated. With the exception of distinguishing adenoma and T2D, all classifiers achieved an AUC greater than 0.7 (Figure 6C). The classification of CRC and adenoma yielded an AUC of 0.73. The classification of diarrhoea from other diseases consistently achieved an AUC no less than 0.97. The potential of HGTs to differentiate between different diseases proved to be unexpectedly promising.

Moreover, the integration of HGT and microbial-abundance biomarkers yielded enhanced predictive performance for CRC. Thomas *et al.* showed that 16 microbial-abundance biomarkers (‘Thomas-Abun’) allowed satisfied CRC prediction performance, and using all remaining species afforded little improvement (43). We attempted to assess whether integrating HGT biomarkers with microbial-abundance biomarkers could improve the predictive performance for CRC. The 16 most differential HGT genus pairs between CRC and controls were selected. To enhance the biomarker repertoire, the genera associated with these genus pairs were combined with the ‘Thomas-Abun’ biomarkers, resulting in a total of 34 microbial-abundance biomarkers. The integration of these 16 HGT biomarkers and 34 microbial-abundance biomarkers formed the ‘Hybrid’ biomarker set. In the LODO validation of eight CRC cohorts, ‘Thomas-Abun’ achieved an AUC of 0.81 on average, and ‘Hybrid’ increased the average AUC to 0.87 (Figure 6D). The AUC of ‘Hybrid’ was higher than ‘Thomas-Abun’ in every validated cohort of the LODO analysis. When using the 16 HGT biomarkers independently, an average AUC of 0.78 was obtained. Moreover, an additional independent CRC cohort (YangJ_2020) of 95 CRC patients and 69 controls validated the excellent performance of ‘Hybrid’ (Figure 6E). ‘Hybrid’ performed better (AUC: 0.87) than ‘Thomas-Abun’ (AUC: 0.85) in the independent CRC cohort. Additionally, a T2D cohort (KarlssonFH_2013) of 140 non-CRC samples validated the CRC specificity of ‘Hybrid’ (Figure 6F). The false positive rate of ‘Hybrid’ (5.0%) was lower than ‘Thomas-Abun’ (9.3%). The integration of microbial-abundance and HGT biomarkers allowed a better CRC prediction performance. Altogether, our results suggested that HGTs within the gut microbiome have the potential to serve as reliable biomarkers to predict human diseases.

### Important bacteria in HGT networks associated with human diseases

The populational HGT network analyses exhibited the important bacteria associated with human diseases. Additionally, the individual HGT network can reflected the gut microbiome alteration for various diseases. Moreover, the scale-free individual HGT network has a higher frequency at lower taxonomic ranks.

**Figure 7.**
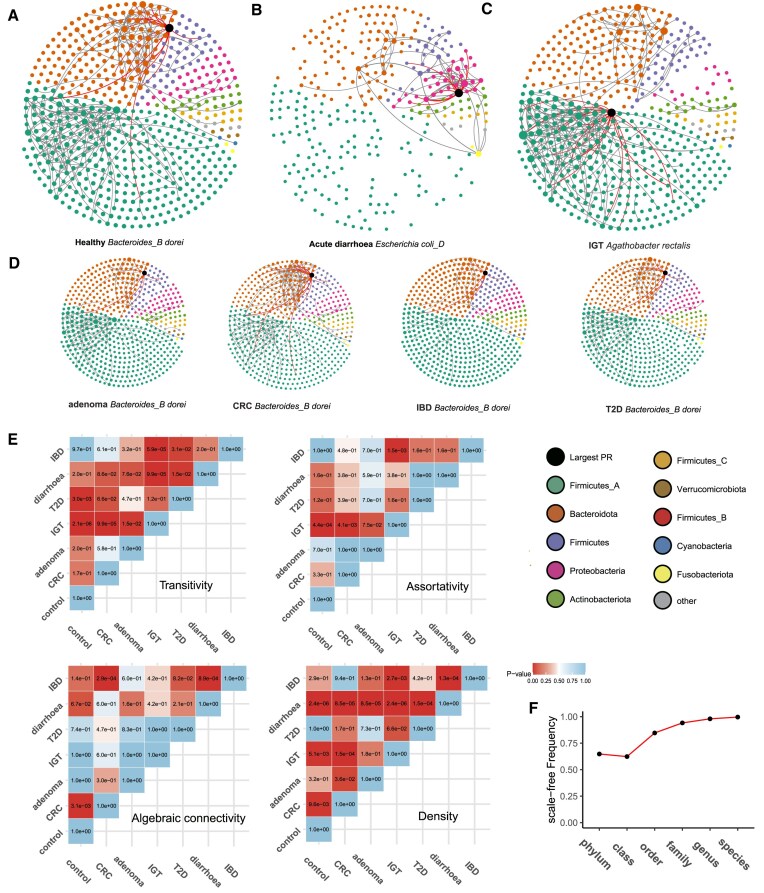
HGT networks are associated with human diseases. (**A–D**) Populational HGT network of different sample groups. Nodes are species, and edges represent the frequency of HGTs between two species in the population. Edges with a frequency less than 0.1 are hidden and the width of the edges shows the frequency. Node colors represent the phylum of the species, node size represents the PR score of each species. The black node represents the species with the highest PR score, and its name is given below the graph. Edges linked to the node with the largest PR score are marked in red. Only the top 600 species with highest HGT frquency are shown. The node with the same relative coordinates between different graphs represents the same species. (**E**) Comparison of individual HGT network properties between different sample groups. The color of each cell indicates the *P*-value for the comparison. (**F**) Frequencies of scale-free individual HGT networks at each taxonomic level.

Analyses of the populational HGT network exhibited important bacteria associated with diseases. A previous study has shown that the HGT network formed by HGT events of gut microbiome is associated with host status (45). We were inspired to explore the relationship between HGT networks and human diseases. For each sample group, we constructed a populational HGT network, where each species is a node, and edges represent the frequency of the HGT species pair in the population (Methods). Important nodes were selected using the PageRank (PR) algorithm, with the PR score measuring the importance of nodes. The populational HGT network structure was similar among controls, CRC, adenoma, IGT, T2D, and IBD (Figure 7A–D). For these groups, the nodes with the highest PR scores mainly belonged to the phyla Firmicutes_A and Bacteroidota. As shown in the above results, the two phyla also had the highest HGT frequency in the population. However, for diarrhoea, the nodes with the highest PR scores were enriched in the phylum Proteobacteria, which has been reported as a pathogenic risk factor for diarrhoea (63). For all the groups except for diarrhoea and IGT, the most important node was *Bacteroides_B dorei*, which was the second species most frequently involved with HGT. In diarrhoea, the most important node was *E. coli_D*, followed by *Escherichia albertii*. The vital role of *E. coli* and *E. albertii* for diarrhoea has been well characterized by previous studies (59,60). *Agathobacter rectalis* was the most important node for IGT, followed by *Agathobacter faecis*, implying the potential association between the two bacteria and IGT, which has not been reported by any previous study.

Furthermore, the individual HGT network analyses implied the gut microbiome alteration for various diseases. For each sample, we constructed an individual HGT network, where each node is a phylum, and the edge indicates the existence of HGT between the two phyla (Methods). After normalization, there were 724, 392, 48, 49, 93, 45 and 122 individual networks constructed for controls, CRC, adenoma, IGT, T2D, diarrhoea and IBD, respectively. Each network comprised 10 edges. Subsequently, we conducted a comparison of network properties between each pair of groups using the Wilcoxon rank-sum test. The *P*-values were corrected using the FDR (Benjamini-Hochberg) method to reduce false positive discovery rate. The topology properties of individual HGT network showed significant differences between various groups (Figure 7E). Compared to controls, IGT exhibited significantly different transitivity (FDR-corrected *P*-value = 2.1e-6) and assortativity (FDR-corrected *P*-value = 4.4e-4). Diarrhoea had a statistically higher density than control, CRC, adenoma, IGT, and T2D. CRC had significantly lower algebraic connectivity (FDR-corrected *P*-value = 3.1e-3) and higher density (FDR-corrected *P*-value = 9.6e-3) than healthy controls. There was no difference between adenoma and controls, which reinforces previous findings that adenoma’s gut microbiome closely resembles healthy people’s (43,64,65). The individual HGT network analyses suggested the gut microbiome alteration of IGT, T2D, acute diarrhoea, IBD and CRC compared to healthy controls. The gut microbiome alterations associated with these diseases have been previously reported (43,66–68). The variance of the gut microbiome between different diseases has also been shown by individual HGT networks (Figure 7E).

The frequency of the scale-free individual HGT networks was observed to be higher at lower taxonomic ranks. Assume a network is scale-free if its degree distribution follows a power law distribution (Methods). We calculated the frequency of the scale-free individual HGT network by dividing the number of scale-free individual HGT networks by the total number of individual HGT networks. At the phylum, class, order, family, genus, and species levels, the scale-free network frequency was 64.8%, 62.3%, 84.6%, 94.1%, 98.0% and 99.6%, respectively (Figure 7F). The frequency of scale-free networks increased with the reduction of the taxonomic rank. The high scale-free network frequency at the species level is consistent with the previous research, which has shown that the HGT networks with reference genomes as nodes (approximately species level) are scale-free (45). The high frequency of scale-free HGT networks at the species level implied that some bacteria species had significantly more connections than others, i.e. a subset of bacteria species transfer sequences with diverse distinct bacteria species, highly exceeding average. Overall, our analyses showed that the HGT network provides useful insights to understand human diseases.

## Discussion

Several methods have been developed to identify HGTs from shotgun metagenomic sequencing data. However, these methods have not been widely adopted likely due to various limitations. Firstly, they often require extensive computational resources. For example, MetaCHIP is a pipeline based on metagenomic assembly that infers HGTs from assembled contigs (20). However, metagenomic assembly tends to produce highly-fragmented contigs and is computationally demanding. DaisySuite and LEMON rely on read alignment to a large reference database, which also requires significant computational resources and running time (25,26). Secondly, existing methods may be inconvenient to install and use. Lastly, none of the methods can reliably deduce complete HGT events, including transferred sequences as well as the corresponding deletion and insertion sites in the donor and recipient genomes, respectively. To address these challenges, we developed a new method called LocalHGT, which enables accurate and rapid detection of complete HGT events from shotgun metagenomic sequencing data. For fast HGT detection, we implemented fast fuzzy *k*-mer matching to expedite the process of read alignment. For convenient application, LocalHGT provides a user-friendly environment construction approach using Conda and offers detailed documentation. To detect complete HGT events, we match the HGT breakpoint pairs based on the association of breakpoints from the same event. One advantage of conducting disease association studies based on HGTs is the ability to identify specific functions and even genes that contribute to the association (21). LocalHGT provides an opportunity to systematically investigate HGT events within the microbiome on a large scale, enhancing our understanding of diseases.

HGTs can be an important supplementary biomarker for disease prediction. Disease prediction is essential for treatment. For example, diagnosing cancer at an early stage usually provides the best opportunity to save lives. Machine learning techniques have been used to predict cancers using various genomic biomarkers (69–71). It has been proved that the gut microbial biomarkers have the potential to be well applied in CRC diagnosis (43,72–76). Previous research has shown that the use of 16 microbial-abundance biomarkers enables accurate CRC prediction, and using additional abundance biomarkers is helpless for improvement (43). In this study, we demonstrated that combining HGTs and microbial-abundance biomarkers can improve the predictive performance of CRC. We presume that integrating different types of biomarkers, such as microbial abundance, gene families, HGT events of the gut microbiome, as well as the physiological factors of the human body, could enhance CRC prediction. Moreover, we have shown that HGTs have the potential to serve as biomarkers to predict IGT, diarrhoea and IBD as well as to distinguish between different diseases.

The gut microbiome is a complex ecosystem in which the compositions have frequent interactions, and should be understood as a network rather than a tree. The phylogenetic tree can only depict vertical gene transfer from parent to offspring (77). The HGT-mediated evolutionary relationships should be depicted by a phylogenetic web or network (1,45,78,79). We constructed HGT networks based on HGT events in the gut microbiome. HGT networks exhibited significant topology differences between different host phenotypes, reflecting the alteration of the gut microbiome. Also, using the PageRank algorithm, we identified the most important nodes in the HGT network. The HGT network offers a nascent layer of variability in the gut microbiome, which can facilitate our understanding of gut microbiome.

Sequencing output amount significantly affects HGT detection by influencing computational resource utilization and HGT detection accuracy. Our study shows that LocalHGT’s CPU time increases with sequencing output amount, but its CPU time reduction compared to the traditional method becomes more significant with higher sequencing output amount, demonstrating LocalHGT’s efficiency in HGT detection (Supplementary Note S2 and Supplementary Figure S7). Furthermore, we have conducted an estimation of the expected sequencing output amount required for HGT detection using LocalHGT (Supplementary Note S2 and Supplementary Figure S8). Our analysis has revealed that a sequencing depth of 30x is generally sufficient for HGT detection within a complex microbial community. By considering the relative abundance of a species in the community, we can suggest an appropriate sequencing output amount for HGT detection specific to that species. Let’s consider a species present in a microbiome sample, with its genome length denoted as *L* and its relative abundance represented as α. To identify HGT within this species, the expected sequencing output amount (number of DNA bases) for the microbiome sample can be calculated using the formula: 30*L/α. For example, if we aim to detect HGTs for a species with a genome length of 3M and a relative abundance of 1%, the sequencing output amount required for the microbiome sample would be approximately 9G. Users can estimate the relative abundance of each species through 16S rRNA sequencing and determine a suitable sequencing output amount for HGT detection in shotgun sequencing.

The performance of downstream analysis methods can be influenced by wet lab procedures in sequencing. For instance, the selection of a library construction kit can impact the accuracy of taxonomic abundance recovery, as demonstrated by higher accuracy in certain kits (80). In our analyzed cohorts, diverse physical treatments, DNA extraction methods, and library construction techniques were employed (Supplementary Table S5). Evaluating the impact of wet lab procedures on HGT detection in these cohorts is challenging due to potential variations in the frequencies of HGT events among different cohorts. For instance, the cohorts WirbelJ_2018 and YachidaS_2019 showed significant differences in the number of detected HGT events, despite having similar sequencing depth and employing comparable wet lab procedures (Supplementary Figure S15). Therefore, it is essential to conduct systematic experiments to thoroughly assess the effects of various wet lab factors on the performance of HGT detection. Also, wet lab factors have the potential to influence the reliability of the identified HGT biomarkers for diseases. Before clinical implementation of these HGT biomarkers, it is essential to perform thorough validation methods, such as qPCR, to ensure their reliability and accuracy.

Our study has several limitations. First, LocalHGT cannot detect HGT events between the different strains of the same microbial species. Since HGTs tend to occur between closely-related genomes, there might be extensive HGTs between the members of the same species, which could provide bacteria with a wide range of functions to adapt to the environment. We intend to deduce intra-species HGTs in the future. Also, we cannot distinguish ancient and recent HGTs using LocalHGT. LocalHGT detects inter-species sequence transfer between the reference genomes, the inferred HGTs are a mixture of ancient HGTs inherited from other places and recent HGTs that occurred in the individual. While both ancient and recent HGTs manifest as genomic features at the strain level, discerning between ancient and recent HGT events can offer valuable insights into the microbial adaptation to the host environment.

## Supplementary Material

gkae515_Supplemental_Files

## Data Availability

The software package LocalHGT is available at https://github.com/deepomicslab/LocalHGT (permanent DOI: https://doi.org/10.5281/zenodo.10995452). The scripts to analyze the HGTs are also in this repository. The raw sequencing data are available in the NCBI database under the following accession numbers: ERP005534 for ZellerG_2014 (65); PRJEB10878 for YuJ_2015 (72); ERP008729 for FengQ_2015 (64); SRP136711 for ThomasAM_2018a and ThomasAM_2018b (43); DRA006684 for YachidaS_2019 (81); PRJEB27928 for WirbelJ_2018 (73); PRJEB12449 for VogtmannE_2016 (82); SRP128485 for YangJ_2020 (74); PRJEB1786 for KarlssonFH_2013 (83); PRJNA422434 for QinJ_2012 (84); ERP002061 for NielsenHB_2014 (85); PRJNA385949 for HallAB_2017 (86); PRJEB9150 for DavidLA_2015 (87); PRJNA363003 for KieserS_2018 (88); and SRP366030 for cross-sectional and time-series cohorts (22). The information of each sample is listed in Supplementary Table S1.
