# Correction to ‘Enhancing insights into diseases through horizontal gene transfer event detection from gut microbiome’

**DOI:** 10.1093/nar/gkaf631

**Published:** 2025-06-23

This is a correction to: Shuai Wang, Yiqi Jiang, Lijia Che, Ruo Han Wang, Shuai Cheng Li, Enhancing insights into diseases through horizontal gene transfer event detection from gut microbiome, *Nucleic Acids Research*, Volume 52, Issue 14, 12 August 2024, Page e61, https://doi.org/10.1093/nar/gkae515

In the originally published version of this paper there was an error in the Funding grant number from the Shenzhen Science and Technology Program. This was incorrectly given as “20220814183301001” instead of “JCYJ20220818101201004”. This error has been corrected.

