# Supplementary material for: Enhancing insights into diseases through horizontal gene transfer event detection from gut microbiome
Source: Nucleic Acids Res. 2024 Jun 17;52(14):e61. doi: 10.1093/nar/gkae515 (PMC11317153; doi:10.1093/nar/gkae515)
Supplement: gkae515_Supplemental_Files [file gkae515_supplemental_files.zip › HGT_Supplemental_Information_R1_clean.pdf]

Supplemental Note

Note S1 Assessment of hash collision during *k*-mer counting

The devised hash function scheme effectively handles collision instances during *k*-mer counting. The empty ratio of the *k*-mer counter array is an assessment metric of the hash collision during *k*-mer counting. The *k*-mer counter array is initialized with zero values before *k*-mer counting. We calculated the empty ratio of the counter array after *k*-mer counting in a metagenomic sample with two approaches. The first approach is to compute the ratio of loci with zero values in the array after *k*-mer counting. Because the sequencing error would give rise to noisy *k*-mers, the second approach is to calculate the ratio of loci with count values smaller than three to reduce the impact of noisy *k*-mers.

We randomly selected 2G base pairs of sequencing read from the Critical Assessment of Metagenome Interpretation (CAMI) high complexity sample. The *k*-mer counting was performed with the length of *k*-mer ranging from 20 to 32. Then we calculated the two types of empty ratio with different lengths of *k*-mer (Figure S2). We found that the empty ratio increased with the length of *k*-mer raising. The increasing tendency was more significant with a *k*-mer length longer than 28. The ratio of loci with a count smaller than three reached 85.7% when *k* reaches 32. The ratio of zero count loci was 55.7% with *32*-mers. The high empty ratio of the counter array could imply a low probability of hash collision, which guarantees the *k*-mer count saved in the array is precise. The number of *k*-mers tend to be low in low-complexity samples; thus, the empty ratio would be higher in low-complexity samples than in high-complexity samples. The results suggested that we can ensure a low probability of hash collision in complex metagenomic data using *32*-mers.

Note S2 Evaluation of the HGT detection method

LocalHGT is reliable for HGT event inference Both real and simulated datasets were utilized to evaluate the performance of LocalHGT in inferring complete HGT events. We adopted Nanopore long reads to validate the inferred HGT event on 200 samples that had both Illumina short-read and Nanopore long-read data available from the cross-sectional and time-series cohorts [1]. To validate each HGT event, we generated a local genome by combining the 100 bp sequence at the head side of the breakpoint on the recipient, the transferred sequence, and the 100 bp sequence at the tail side of the breakpoint on the recipient. Then the long reads were aligned to the local genome using Minimap2 [2]. If the transferred sequence was shorter than 8,000 bp, we considered the HGT event to be verified if the genome could be covered by a long read. If the transferred sequence was longer than 8,000 bp, we defined the HGT event as verified if the merged mapped regions of two long reads could cover the genome. Since some genomes may not have any long reads sequenced due to the limitations of sequencing output, we only examined the HGT events with transferred sequences supported by long reads to ensure a fair validation. LocalHGT demonstrates a remarkable accuracy of 99.4% (4,748 out of 4,775) in the detection of complete HGT events across 200 gut metagenomic samples. This high level of accuracy underscores the reliability of LocalHGT in accurately identifying HGT events.

To further evaluate LocalHGT for HGT event inference, we simulated a dataset with various depths (from 10x to 100x), 10 replicates for each depth, resulting in 100 samples. We then ran LocalHGT to match HGT breakpoints to infer HGT events. Subsequently, we calculated the F1 score of HGT event inference for each depth threshold (Figure S4). LocalHGT achieved an average F1 score of 0.99 for complete HGT event detection. These results suggested that LocalHGT was reliable for HGT event inference.

LocalHGT is accurate in HGT breakpoint detection LocalHGT demonstrated robustness in detecting HGT breakpoints across different sequencing depths and variations in the divergence between the local genome and the reference. We first sought to determine whether LocalHGT could be well applied in datasets with different depths. Eleven datasets with depths ranging from 5x to 100x were generated, and each dataset contains ten replicates. In each replicate, we simulated 20 genomes and 20 HGT events. We ran LocalHGT and LEMON on the datasets with default parameters and ten threads. We assessed the HGT breakpoint detection performance of LocalHGT and LEMON using the F1 score, which balances the recall and precision (Figure S5). The mean F1 scores of LocalHGT were slightly higher than LEMON when the depth was higher or equal to 10x, and the average F1 scores of both tools were above 0.98. For the low-depth dataset with 5x depth, the F1 score of LocalHGT was much higher than LEMON, with an average F1 score above 0.93 compared to LEMON's score below 0.86. These results revealed that both LocalHGT and LEMON have high accuracy and precision of HGT breakpoint detection, and LocalHGT outperformed LEMON across all the depths, especially at the low depth (5x).

Next, we investigated how the divergence of the local genome and reference affects the performance of LocalHGT. Local bacterial genomes might diverge from the reference representative genomes, and this divergence could potentially harm LocalHGT's performance. We simulated genomes with different mutation rates. We generated five datasets with mutation rates ranging from 0.01/0.001 to 0.05/0.005 (the former value

is the mutation rate of SNVs, and the latter is for InDels). The mutation rate of SNVs is ten times higher than InDels. Ten replicates were generated for each value of mutation rate with 30x sequencing depth. We then performed LocalHGT and LEMON on the datasets and measured their performance with the F1 score (Figure S5). Both tools achieved mean F1 scores above 0.97 across different mutation rates. The F1 scores did not decrease as the mutation rate increased, indicating that LocalHGT is robust against the genome divergence.

LocalHGT is fast in complex metagenomic samples LocalHGT demonstrates notable efficiency in the analysis of complex metagenomic samples, with its efficiency being particularly notable when handling large reference databases. We evaluated the accuracy and efficiency of LocalHGT on complex metagenomic samples. To simulate complex metagenomic samples, we generated five samples with varying SNV/INDEL mutation rates from 0.01/0.001 to 0.05/0.005. The Illumina paired-end reads were generated with 100x depth and 150 bp read length. The five simulated samples were combined with CAMI samples with varied complexities (Low, Medium, High), resulting in 15 metagenomic samples with different mutation rates and complexities. LocalHGT and LEMON were used to identify HGT breakpoints based on the UHGG reference [3]. We executed LocalHGT and LEMON with default parameters and ten threads. Recall was used to assess the HGT breakpoint detection accuracy in complex samples, as unknown HGT events might exist in CAMI datasets. The CPU time and Peak RAM were used to assess the computational requirement of both tools (Figure S6). The recall was 100% for both tools in all the samples, suggesting high HGT breakpoint detection accuracy of LocalHGT and LEMON in complex metagenomic samples. LocalHGT's speed advantage is more significant in low-complexity samples than in high-complexity samples. On average, LocalHGT processed a complex microbiome sample with 1.7 hours wall-clock time, 7.3 hours CPU time and 21.7 G peak memory and LEMON cost 6.5 hours wall-clock time, 42.1 hours CPU time, and 22.3 G peak memory. Compared to LEMON, LocalHGT consumed 73.8% less wall-clock time, 82.7% less CPU time, and 2.7% less peak memory relatively, on average. Furthermore, the computational consumption associated with matching breakpoint pairs for event inference can be considered negligible. These findings strongly suggest that LocalHGT achieves a high level of efficiency in detecting HGT events.

LocalHGT's advantage becomes increasingly evident and amplified when confronted with large reference databases. To assess the impact of a larger reference database, we downloaded the proGenomes v2.1 representative database, which contains representative genomes for all bacteria. The size of proGenomes v2.1 is 45.4G, which is much larger than the UHGG database (11.4G). We ran LEMON and LocalHGT on a sample (SRA ID: SRR6915229) with the proGenomes v2.1 as the reference database. The peak RAM values of LEMON and LocalHGT were 79.0G and 22.7G, respectively, and the CPU time values of the two methods were 18.8 hours and 7.5 hours, respectively. LocalHGT was not only faster than LEMON but also required much less memory. LEMON's memory consumption is up to the reference size, while LocalHGT's memory cost mainly depends on the length of k-mer. The results indicated that LocalHGT could rapidly detect HGT events in complex metagenomic data with high accuracy.

Assessing the impact of sequencing output amount on the speed of LocalHGT To assess the impact of sequencing output amount on the computational resource requirements of LocalHGT, we performed random downsampling of the CAMI datasets to generate data with varying sequencing output amounts. The CAMI high, medium, and low complexity datasets contained 14,971,780,500 bp, 14,975,651,700 bp, and 14,969,453,700 bp DNA bases, respectively. For each dataset, we randomly selected reads at sampling fractions ranging from 0.1 to 1.0, incremented by 0.1, resulting in 30 samples with diverse complexities and sequencing output amounts. Subsequently, we executed LocalHGT and LEMON on these samples using ten threads and default parameters. We recorded the CPU time and peak RAM consumption for each tool. In all samples, LocalHGT outperformed LEMON in terms of speed, and the disparity in CPU time between LocalHGT and LEMON increased with larger sequencing output amounts (see Figure S7). The CPU time increased as the sequencing output amount increased for both LocalHGT and LEMON. However, the peak RAM usage of both tools did not show significant variation with changes in sequencing output amount. These results demonstrate that LocalHGT exhibits swiftness in detecting HGT from complex metagenomic data, with its speed advantage becoming more pronounced as the sequencing output amount grows.

Estimating the expected sequencing output amount required by LocalHGT The microbiome sample contains numerous species with very low abundance (less than 0.1%), making it challenging to achieve sufficient sequencing depth for all species to detect HGTs, even with ultra-deep sequencing. To address this issue, we estimated the expected sequencing depth required by LocalHGT for each species. Based on the expected sequencing depth and the relative abundance of each species in the community, we can determine the necessary amount of sequencing output for a given sample. In our study, we focused on an ultra-deep gut microbiome sequencing sample of 753.7G base pairs (sample name SAMEA5669781, accession code PRJEB24152) [4]. We performed subsampling on the ultra-deep sequencing sample, generating ten samples

with increasing sequencing data amounts ranging from 10G to 100G (incremented by 10G). To analyze the species abundances of the subsampled samples, we utilized Kraken2 [5]. As the amount of sequencing data increased, the number of species detected by Kraken2 also increased (Figure S8a). In the 100G sample, we found that 514 species exhibited very low abundance (less than 0.1%) (Figure S8b). Notably, even with 100G base pairs of data, 233 species had extremely low sequencing depth (less than 5x) (Figure S8c). This indicates that even with a high amount of sequencing data, many species still exhibit a very low sequencing depth due to their low abundance in the microbial community.

The analysis of the real microbiome data revealed that a sequencing depth of 30x is the expected requirement for the genome targeted by LocalHGT. We proceeded to run LocalHGT on the ten subsampled samples, calculating the genome depth and the number of related HGT breakpoint pairs for each genome. By examining the number of HGT breakpoint pairs, we classified the genomes into two categories based on their maximum breakpoint pairs: 5-20 and >20. The genomes with less than 5 breakpoint pairs when the depth exceeded 30x were excluded. With sequencing depth increasing, we visualized the change in the number of detected HGT breakpoint pairs for each category (Figure S8d-e). Although the number of HGT breakpoint pairs increased with increasing sequencing depth, this upward trend was significantly reduced when the depth reached 30x. In this analysis, we excluded species that exhibited a substantial increase in the number of detected HGT breakpoint pairs even with depths exceeding 50x. These species may contain multiple strains [6], and the HGT breakpoints could vary significantly across different strains. Additionally, certain strains may exhibit very low abundance. In order to identify all HGT breakpoints for all strains, a substantially higher sequencing depth of the genome is necessary. Consequently, the number of detected HGT breakpoints would not converge within 50x sequencing depth. The results demonstrated that a sequencing depth of 30x is suitable for LocalHGT analysis in the real data. Additionally, when we classified the genomes into two groups based on their length, $> 3M$ and $\leq 3M$, we observed that although longer genomes tended to have a higher number of detected HGT breakpoint pairs, the two groups of genomes all converged at approximate 30x depth (Figure S8f).

The estimation of the required sequencing output amount for LocalHGT can be based on the expected sequencing depth of each species. Let's consider a species within a microbiome sample, where the length of the species genome is denoted as L and the relative abundance of the species is represented by α. To identify HGT breakpoints in this species, the expected sequencing output amount for the microbiome sample can be calculated as $30 * L/\alpha$. For instance, in order to detect HGTs for a species with a length of 3M and a relative abundance of 1%, the estimated sequencing output amount required for the microbiome sample would be approximately 9G. In practical scenarios, it may be necessary to conduct 16S rRNA sequencing to estimate the relative abundance of each species. By obtaining this information, we can then estimate the expected sequencing output amount required for HGT detection in shotgun sequencing.

LocalHGT is robust with the absence of donor genome LocalHGT exhibits resilience in the face of donor genome's status. To test if the donor genome impacts the performance of LocalHGT, we simulated two datasets with different statuses of the donor genome. We randomly selected five donor genomes and five recipient genomes for each sample. Then we randomly chose fragments from the donor genomes and inserted them into the recipient genome. We utilized donor and recipient genomes to simulate sequencing reads in the first dataset and discarded the donor genome in the second dataset. Ten replicates were generated in each dataset. Then we ran LocalHGT and LEMON in these two datasets and compared their HGT breakpoint detection accuracies. We found that both tools precisely identify HGTs in these two datasets, with the F1 score equal to 1 in all the samples (Figure S9a). The results showed that the presence and absence of the donor genome does not impact LocalHGT.

Evaluation of LocalHGT with different insert sizes LocalHGT is robust with various insert sizes of sequencing reads. To evaluate LocalHGT's robustness against insert size, we simulated paired-end reads using varying insert sizes ranging from 200 to 950 bp. Ten replicates were generated for each value of insert size. 20 HGT events were simulated in each sample. We simulated paired-end reads with different insert sizes using the parameter *–mflen* of ART_Illumina V2.5.8 [7]. The standard deviation of insert size was set as 10. The sequencing read length was 150 bp, and the sequencing depth was 30x. We ran LocalHGT and LEMON on the samples and compared their HGT breakpoint detection accuracies with different insert sizes. We observed that the average F1 scores of both tools were higher than 0.98 with insert sizes equal to or larger than 350 bp (Figure S9b). In the samples with insert size equivalent to 200 bp, the mean F1 score of LocalHGT was much higher than LEMON. The results indicated that LocalHGT was more robust than LEMON with the short insert size, and LocalHGT could be well applied to samples with different insert sizes.

LocalHGT is robust with short sequencing reads LocalHGT demonstrates robustness when handling short sequencing reads. To assess LocalHGT's performance with a short read length, we simulated ten replicates with a read length of 75 bp. Then we compared the HGT breakpoint detection accuracy of LocalHGT

and LEMON on this dataset (Figure S9c). The F1 score values of both tools were equal to or higher than 0.95. The median F1 score value of LocalHGT was higher than LEMON. The result demonstrated that LocalHGT could perform accurately with a short read length.

LocalHGT enables efficient multi-parallel processing To assess LocalHGT's run time with different numbers of threads, we performed LocalHGT on the CAMI high complexity sample with the number of threads ranging from 2 to 20. We first used two threads to run LocalHGT and added two threads each time until the number of threads reached 20. We ran each experiment three times and computed the mean wall-clock time. The wall-clock time decreased with the number of threads increasing (Figure S9d). The decreased trend became less significant with more than ten threads. With four threads, LocalHGT finished running a sample in less than 3 hours. With 20 threads, the wall clock time was lower than 1.5 hours. The result suggested that LocalHGT can detect HGT in a multi-thread manner.

Construction of the microbiome reference We adopted a gut-specific reference for HGT detection in the gut metagenomic data. We collected the representative genomes of 4,644 gut prokaryotes from the Unified Human Gastrointestinal Genomes (UHGG) collection as of December 2020 [3], and merged them into a single reference file in *fasta* format. The gut-specific UHGG database has a size of 11.4G. For efficiency consideration, we generate the hash functions and compute the hash values for all the *k*-mers on the reference and store them in an index file. To test the memory consumption of LocalHGT with a large reference, we also downloaded the proGenomes v2.1 database (`https://progenomes.embl.de/index.cgi`), which contains the representative genome for every species cluster, not limiting habitat-specific species. The size of the proGenomes v2.1 database is 45.4G. Users can collect the representative genomes for concerned prokaryotes and construct a custom reference database to run the LocalHGT software.

Simulation of metagenomic data To evaluate the accuracy and efficiency of LocalHGT in detecting HGT, we simulated samples with artificial HGT events. For each sample, forty genomes were randomly selected from the UHGG database, with twenty serving as donors and the other twenty as recipients. To avoid simulating HGT events at the homology genes, we aligned the UHGG database onto itself using Blastn and collected the unique sequence intervals. Then we randomly chose the insert position in the recipient genome and the transferred segments in the donor genome from the unique sequence intervals. The length of the transferred segments was randomly selected between 500 bp and 5,500 bp. The transferred segments were inserted into the recipient genomes at the insert positions.

Unless otherwise specified, we simulated sequencing reads as follows. The donor genomes were discarded, and the inserted recipient genomes were adopted to simulate sequencing reads. We simulated variants on the genome by randomly inserting SNVs and single-nucleotide INDELs into the genomes with a mutation rate of 0.01/0.001 (0.01 for SNVs and 0.001 for INDELs). We simulated 150 bp paired-end reads of 100x depth in each sample. ART_Illumina V2.5.8 was performed with the command *art_illumina -ss HS25 -nf 0 –noALN -p -l 150 -m 350 -s 10 –fcov 30 -i genome -o prefix* to simulate reads with Illumina characteristics [7].

We generated samples with different conditions to evaluate LocalHGT. To assess LocalHGT in different depths, we simulated samples with eleven levels of depth: 5x, 10x, 20x, 30x, 40x, 50x, 60x, 70x, 80x, 90x, and 100x. We also simulated samples with five levels of mutation rates from 0.01/0.001 to 0.05/0.005. To evaluate LocalHGT with a short read length, we generated paired-end reads with a length of 75 bp. In addition, we simulated samples with or without donor genomes to evaluate the influence of the donor genome on LocalHGT. With each parameter combination, we generated ten replicates.

We simulated complex metagenomic samples by merging the pure samples and CAMI datasets. We generated five pure samples with different levels of mutation rate (from 0.01/0.001 to 0.05/0.005). We then combined pure samples with CAMI datasets at varying complexities (low: RL_S001__insert_270, medium: RM2_S001__insert_270, high: RH_S001__insert_270) [8]. The CAMI datasets were obtained from `https://edwards.sdsu.edu/CAMI/`. Fifteen complex metagenomic samples with varying mutation rates and complexities were obtained. We adopted these samples to test the efficiency and accuracy of LocalHGT in metagenomic samples. We compared LocalHGT with LEMON in benchmark datasets. DaisySuite was discarded since it took more than a day to handle a sample.

Evaluation metrics The recall is a measure of the fraction of HGT events that were correctly identified out of the total number of HGT events that were simulated. Precision, on the other hand, is a measure of the fraction of HGT events that were correctly identified out of the total number of identified HGT events. To combine both recall and precision into a single score, the F1-score is computed using the formula:

$$F_1 = \frac{2}{\frac{1}{recall} + \frac{1}{precision}}. \tag{1}$$

F1-score is a useful measure to assess the accuracy of HGT breakpoint detection in pure simulated samples. However, since the ground truth of HGT events in the CAMI datasets is unknown, only recall is used as a metric in evaluating the accuracy of LocalHGT in complex metagenomic samples.

The computational cost of the HGT detection process is also evaluated. Two measures are used for this purpose: CPU time and Peak RAM. Most of the comparisons were performed on a personal computer with 12th Gen Intel(R) Core(TM) i7-12700F processor (2.10Ghz, 12 cores) and 32 GB RAM. The experiments with the proGenomes v2.1 as the reference were performed on a server with Intel(R) Xeon(R) CPU E7-4850 v2 processor (2.30GHz, 12 cores) and a total of 598 GB global shared memory. To compute the CPU time, the user time and system time are summed up. Peak RAM, on the other hand, is the maximum resident set size that is reported by the Linux command *time -v* while executing the method. Since the computational resource consumption for matching HGT breakpoint pairs in complete HGT event detection is significantly smaller compared to HGT breakpoint detection, we focused solely on evaluating the computational resource consumption of HGT breakpoint detection.

Parameter adjustment LocalHGT's hyper-parameters can be adjusted to achieve various goals efficiently. The tool can detect HGT given any reference composed of representative genomes, although it is recommended to use the environment-specific reference to save computational resources. When using a small reference, the alignment step is not the computational bottleneck, and the HGT-related segment extraction step can be skipped to reduce memory usage of LocalHGT. When using a large reference, increasing the length of *k*-mers can reduce collision probability and improve the sensitivity of HGT detection. The hash collision can negatively impact the sensitivity and efficiency of LocalHGT. For example, the hash collision might reduce the depth change signal around the breakpoints. Also, the collision might cause false-positive HGT breakpoint identification while using *k*-mer markers to identify cross-species breakpoint pairs. To minimize hash collision, the length of *k*-mer can be increased. LocalHGT includes a reference reduction procedure that extracts the reference fragments present in the sample using the ratio of *k*-mer hits. Increasing the cutoff of the *k*-mer hit ratio values can make the HGT-related segments much smaller, but it may also falsely discard genomes highly diverged from the reference. It is necessary to balance the HGT detection sensitivity and the efficiency by adjusting LocalHGT's parameters.

Note S3 Differential involvement of MGEs in inter- and intra-phylum HGT events

MGEs are vital in facilitating HGT events and MGEs might exhibit distinct roles in driving inter- and intra-phylum HGT events. Although rare overall, a substantial number of inter-phylum HGT events were detected. Out of the complete HGT events identified by LocalHGT, 10.3% (1,986/19,343) were found to be inter-phylum. MGEs were frequently associated with both inter- and intra-phylum HGT events. We systematically identified MGEs located in the flanking regions surrounding the HGT events by searching against the ImmeDB database [9] (Methods). In total, 62.0% (1,231/1,986) of the inter-phylum HGT events and 71.0% (12,330/17,357) of the intra-phylum HGT events were associated with MGEs (Figure S11a-b). The result suggested the significant contribution of MGEs in driving HGT processes.

MGEs might play distinct roles in mediating HGTs within and between phyla. MGEs on the donor genome were more commonly involved in intra-phylum HGT events (Fisher's exact test, *P*-value=3.1e-22), whereas MGEs on the recipient genome exhibited a higher frequency in inter-phylum HGT events (Fisher's exact test, *P*-value=1.3e-33). Moreover, significant differences were observed in the frequencies of specific categories of MGEs involved between inter-phylum and intra-phylum HGT events. Genomic islands, group II introns, and prophages accounted for significantly lower proportions in inter-phylum HGT (Fisher's exact test, Bonferroni-corrected P-value$<$0.05). In contrast, transposons were significantly enriched in inter-phylum events (Fisher's exact test, Bonferroni-corrected *P*-value=1.3e-18, Figure S11c). The dependence on specific host factors might limit the ability of prophages, genomic islands/islets, and group II introns to mediate inter-phylum HGT events. Prophages and genomic islands have co-evolved with hosts, acquiring specialized integration mechanisms and dependencies on host factors for regulation [10, 11]. The mobility of group II introns also relies upon host-encoded splicing factors for propagation [12, 13]. The MGE involvement difference observed between intra- and inter-phylum HGT events provides insights into the specialized adaptation of MGEs to propagate genes across different evolutionary distances.

Furthermore, MGEs were found to be abundant in the transferred sequences of both inter- and intra-phylum HGT events, with the categories of MGEs varying between these two types of HGTs. In total, 22.9% (454/1,986) and 29.0% (5,039/17,357) of the transferred sequences contained MGEs in inter- and intra-phylum HGT events, respectively. Notably, among MGEs in the transferred sequence, transposons were significantly more abundant in inter-phylum HGT events than intra-phylum HGT events (77.3% vs. 46.8%, Fisher's exact test, *P*-value=1.7e-33, Figure S11d). Remarkably, the transposon NZ_GG703857.1_43343_44503_Transposons305, identified from *Prevotella copri* DSM 18205 in the ImmeDB database [9], constituted 59.0% (268/454) of the MGEs within the transferred sequences involved in inter-phylum HGT events.

The gene transfer into Firmicutes from gram-negative phyla appears independent of MGEs. Interestingly, a significant majority of inter-phylum HGT events involving three specific phylum pairs (Firmicutes-Cyanobacteria, Firmicutes-Euryarchaeota, and Firmicutes-Fusobacteriota) were found to lack neighboring MGEs, accounting for 99.0% (98/99) of these events (Figure S11e). In these HGT events, Firmicutes exclusively served as the recipient. Firmicutes are gram-positive, while the three donor phyla are all gram-negative. Additionally, the gene transfer into Firmicutes from gram-positive phyla involves MGEs. Actinobacteriota, as the only other gram-positive phylum apart from Firmicutes, frequently transfers genes into Firmicutes through MGEs (100%, 25/25). Based on these findings, it appears that gene transfer into Firmicutes from gram-negative phyla is not primarily facilitated by MGEs.

Note S4 HGTs provide insights for understanding human diseases

In addition to colorectal cancer (CRC) and acute diarrhea, HGTs also exhibited functional associations with inflammatory bowel disease (IBD) and impaired glucose tolerance (IGT). Through our analysis, we identified 25 enriched and 195 depleted HGT genus pairs within IBD samples (Figure S15). These genus pairs involved 117 genera, representing 41 families. Notably, 45 of these HGT-involved genera belonged to the family *Lachnospiraceae*. Among the differential genus pairs, the most frequently involved genera were *Agathobacter* (27 occurrences), *Faecalibacterium* (23 occurrences), and *Roseburia* (21 occurrences). It has been reported that *Agathobacter*, a butyrate-producing bacteria, is reduced in patients with ulcerative colitis [14]. KEGG pathway analysis of the differential genus pairs revealed six distinct pathways enriched in IBD-enriched genus pairs and 40 differential pathways in IBD-depleted genus pairs. Notably, the peptidoglycan biosynthesis was identified as the second most significantly enriched pathway within IBD-enriched HGTs. This finding aligns with previous research that observed a pronounced overabundance of the peptidoglycan biosynthesis pathway in patients with ulcerative colitis [15]. Our results suggested a potential association between HGTs and the heightened occurrence of the peptidoglycan biosynthesis pathway in IBD samples.

The comparison between IGT and the control revealed 22 differential HGT genus pairs, with 21 being enriched in IGT and 1 in the control group (Figure S14). These differential genus pairs involved a total of 26 genera, with 8 of them belonging to the family *Lachnospiraceae*. Further KEGG analysis identified 45 differential pathways in the IGT-enriched genus pairs and 9 differential pathways in the IGT-depleted genus pairs. Notably, the ABC transporters pathway exhibited the highest enrichment among the IGT-enriched genus pairs, while it was the most depleted pathway in the IGT-depleted genus pairs. The ABC transporters pathway plays a critical role in glucose and lipid metabolism, which is particularly relevant in the context of T2D [16]. Furthermore, the pathways related to porphyrin metabolism, selenocompound metabolism, and C5-branched dibasic acid metabolism showed enrichment in the IGT-enriched genus pairs, indicating their potential involvement in the observed metabolic dysregulation in individuals with IGT. These findings highlighted the potential role of HGTs in contributing to IGT through the involvement of these pathways. Furthermore, an HGT association analysis was conducted for T2D and adenoma. However, the analysis revealed only three differential genus pairs between T2D and control groups. Notably, no differential genus pairs were identified between adenoma and control groups.

Supplemental Figures

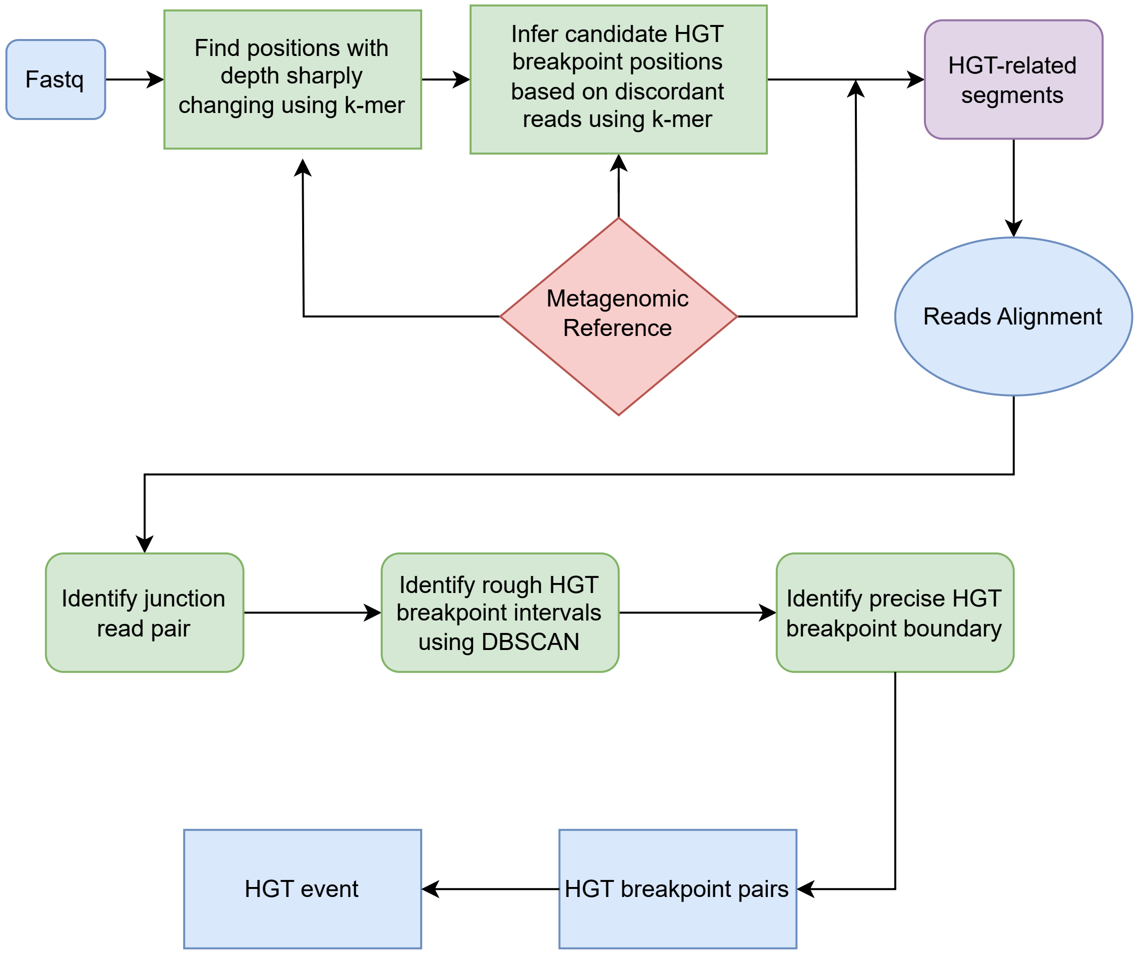

Figure S1: Flowchart of LocalHGT. Given the sequencing reads, LocalHGT first identifies the HGT-related segments using fuzzy *k*-mer matching, and then detects precise HGT breakpoint positions based on reads alignment to the HGT-related segments. Finally, LocalHGT matches HGT breakpoint pairs to identify complete HGT events.

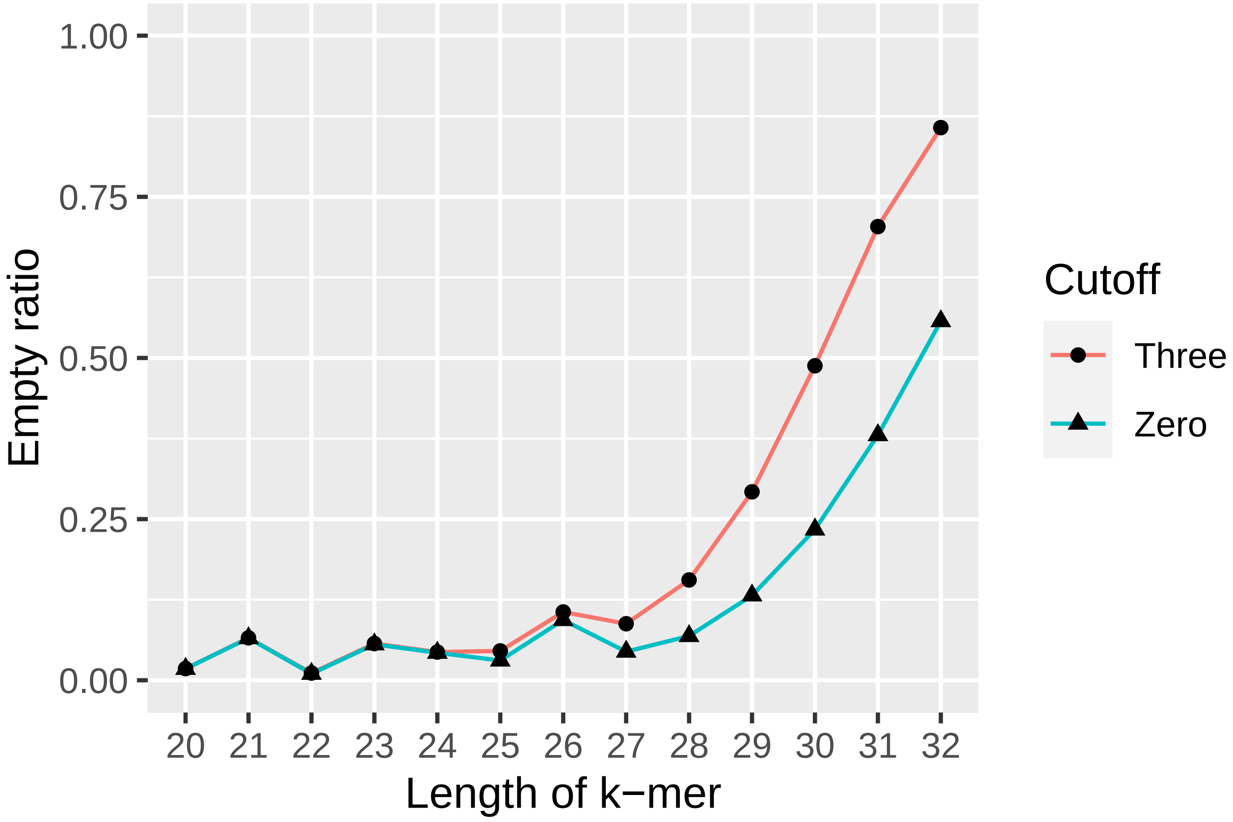

Figure S2: Empty ratio of the hash table after *k*-mer counting. We calculated the empty ratio after *k*-mer counting in the CAMI high complexity sample. The empty ratio was computed with two cutoffs: 'Three' represents the ratio of loci with a count smaller than three, and 'Zero' represents the ratio of positions equal to zero.

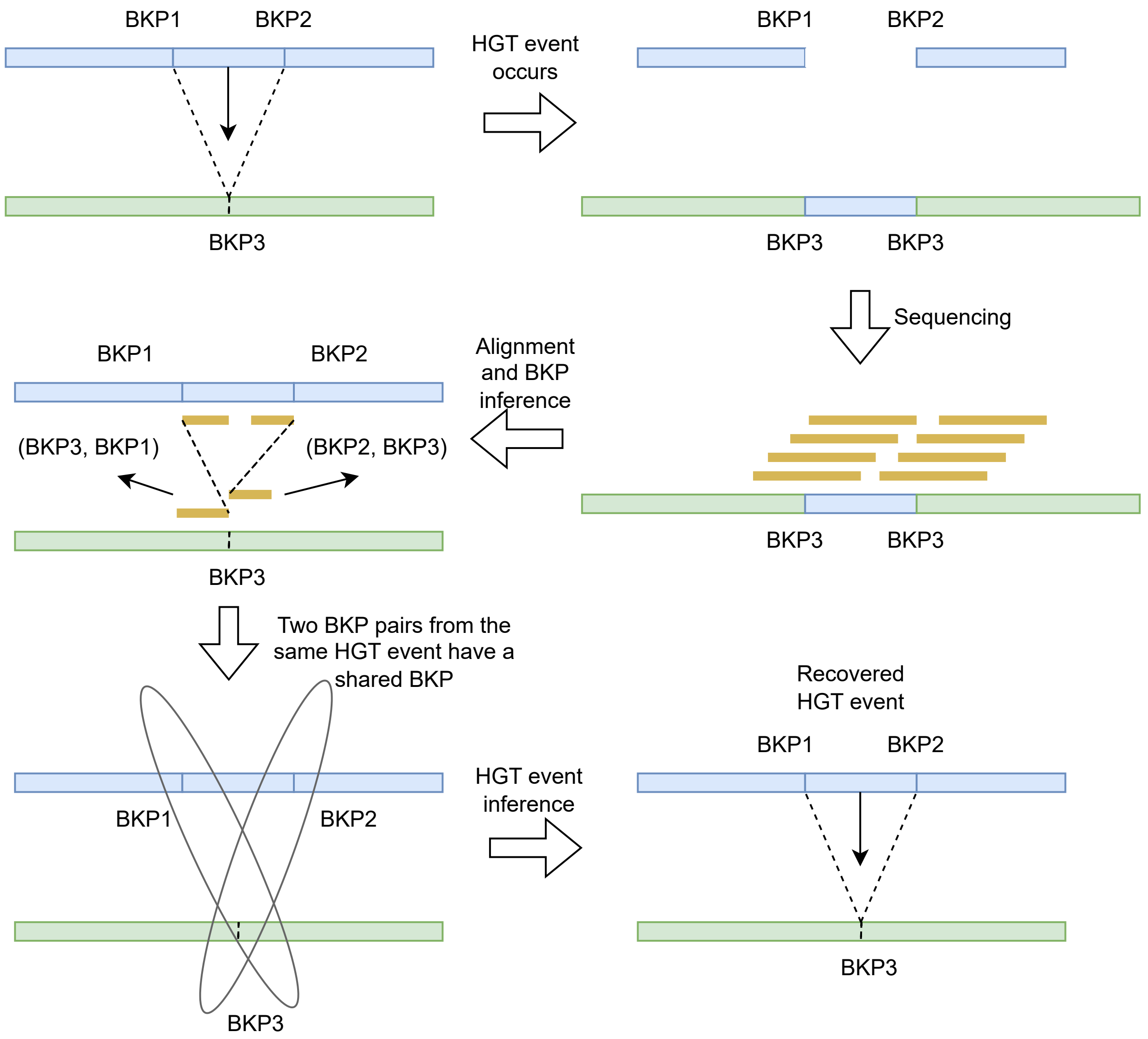

Figure S3: Illustration of inferring the HGT event. After an HGT event occurs between two genomes, we can identify two HGT breakpoint pairs according to the sequencing reads aligned on the two genomes. The two HGT breakpoint pairs derived from the same HGT event have a shared breakpoint, and the other two distinct breakpoints come from the same genome. By matching the two breakpoint pairs, we can recover the original HGT event. 'BKP1', 'BKP2', and 'BKP3' represent HGT breakpoints. The donor genome is denoted by the blue bar, while the green bar signifies the recipient genome. The yellow bars, on the other hand, represent the sequencing reads.

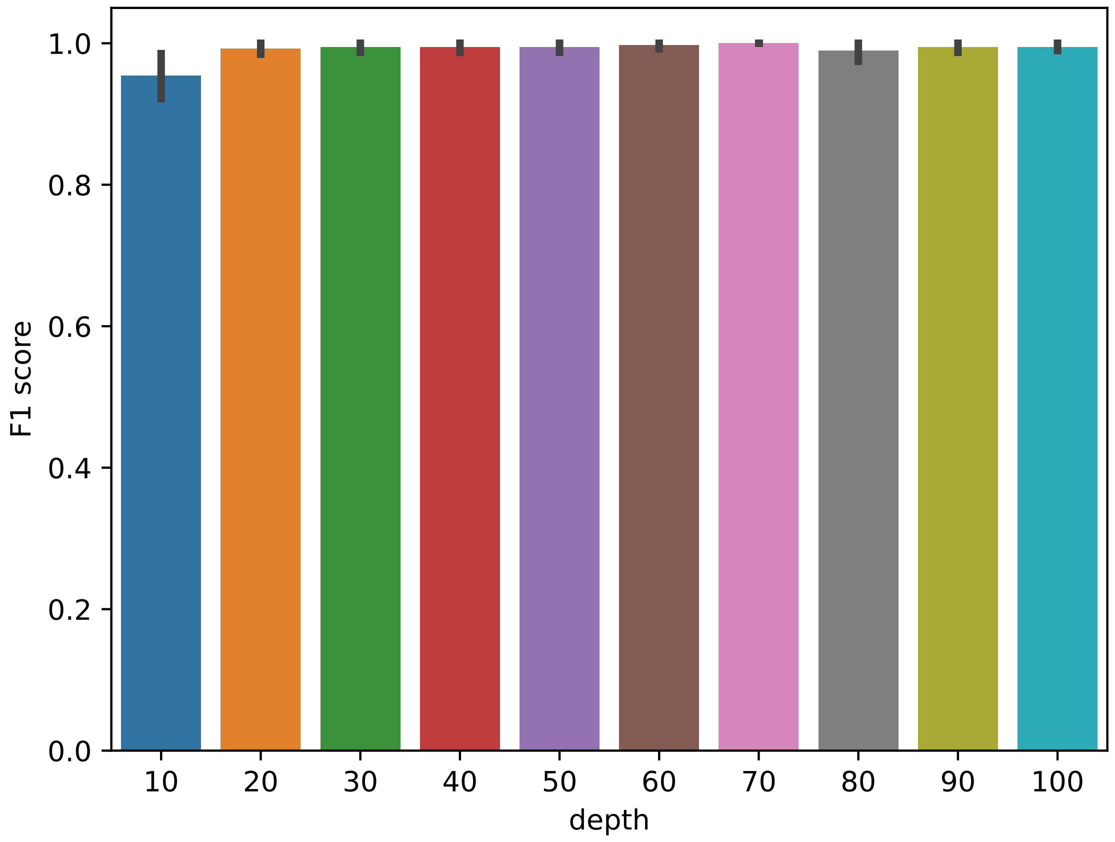

Figure S4: Evaluation of HGT event inference. We performed LocalHGT in the simulated dataset with various depth, and calculated the F1 score of HGT event inference for each depth threshold. Each depth has ten replicates.

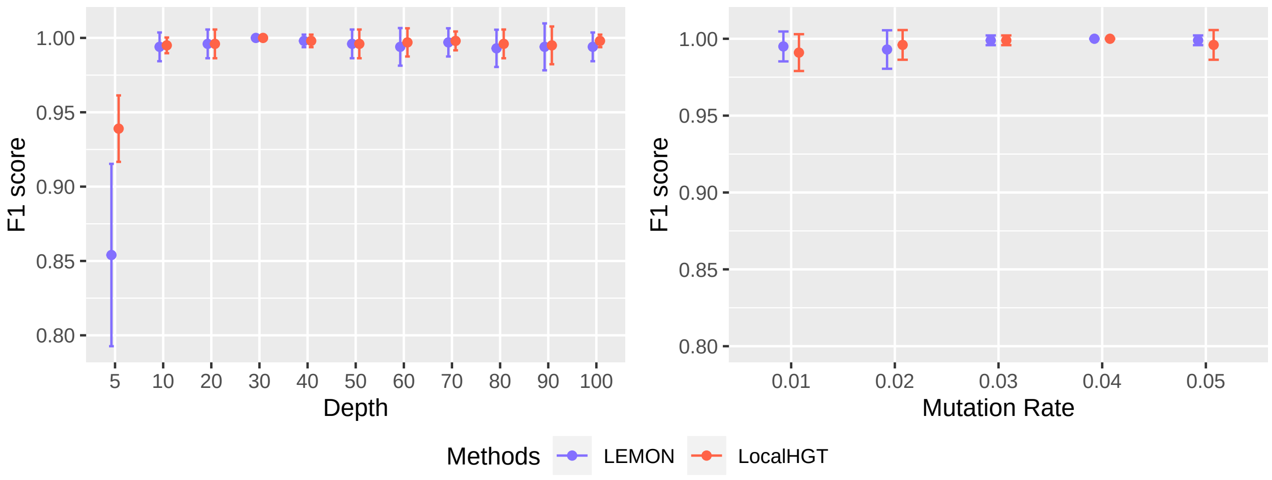

Figure S5: HGT breakpoint detection performance comparison of LocalHGT and LEMON in simulated samples with different depths and mutation rates. The left subfigure shows the comparison in different sequencing depths. The height of the point indicates the mean F1 scores of the replicates, and the error bars represent the standard deviations. We generated HGTs on 20 genomes, simulated reads with different depths, and performed LocalHGT and LEMON on the simulated data. The F1 scores of both tools were larger than 0.9 even with 5x depth in LocalHGT, and the F1 scores of LocalHGT were slightly higher than LEMON in different depth datasets; The right subfigure shows the comparison with different mutation rates. We randomly added variants to the original genomes with mutation rates ranging from 0.01/0.001 to 0.05/0.005. The F1 scores of both tools were higher than 0.97, with different mutation rates.

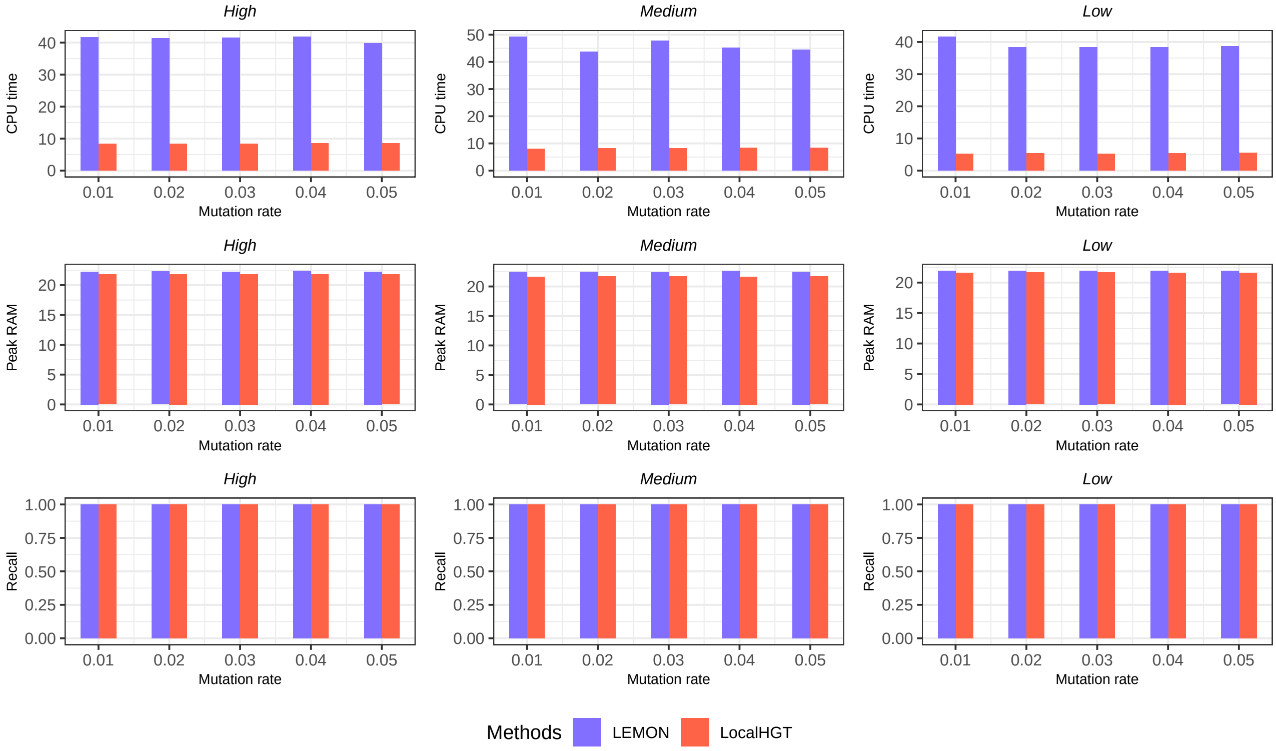

Figure S6: HGT breakpoint detection accuracy and efficiency comparison of LocalHGT and LEMON in complex metagenomic data. (a) To validate LocalHGT's performance on complex metagenomic data, we simulated HGTs on 20 genomes and combined the simulated reads with CAMI datasets with different complexity levels (Low, Medium, and High). The CPU time, peak RAM usage, and recall of LocalHGT and LEMON on these samples are illustrated. The results revealed that LocalHGT required less CPU time than LEMON and achieved comparable HGT breakpoint detection accuracy with LEMON.

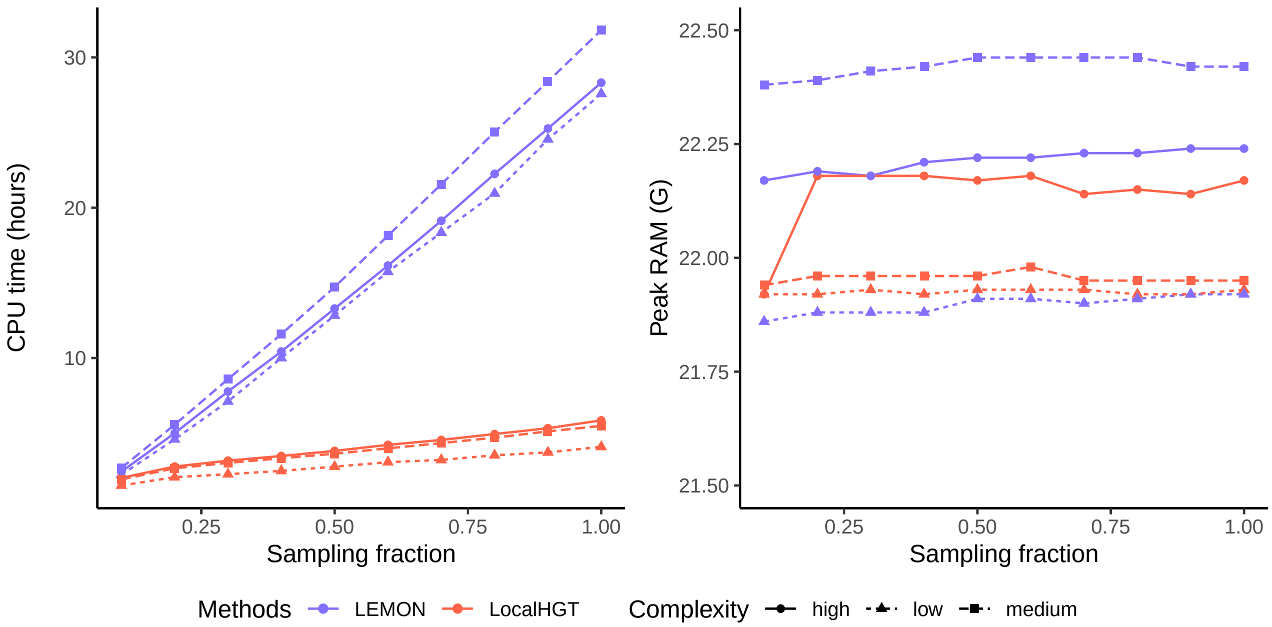

Figure S7: Computational resource assumption of LocalHGT and LEMON with various sequencing output amounts. To generate samples with various sequencing output amounts, we randomly selected sequencing reads from the three CAMI datasets. We used a sampling fraction range of 0.1 to 1.0, resulting in a total of 30 samples. The CPU time (on the left) and peak RAM (on the right) of LEMON and LocalHGT on these samples are illustrated.

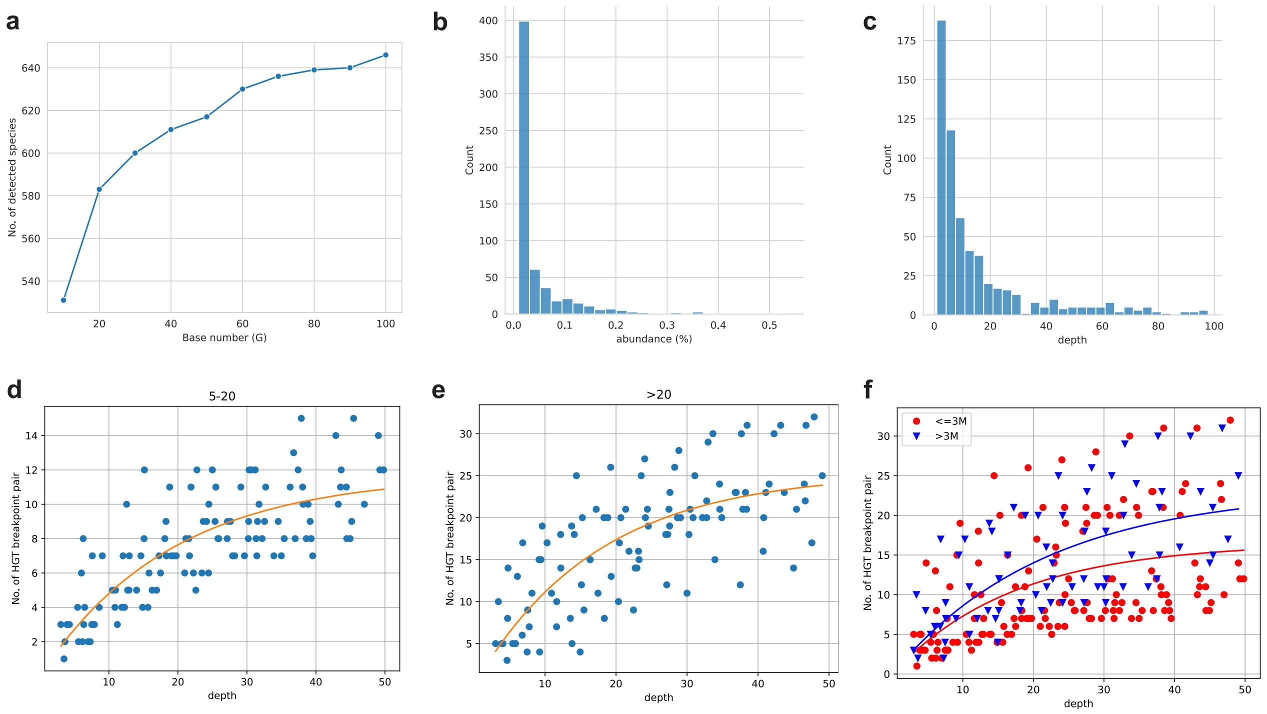

Figure S8: Estimation of the expected sequencing output amount required by LocalHGT. (a) Number of species detected by Kraken2 with increasing sequencing output amount. (b) Abundance distribution of different species. (c) Depth distribution of different species. (d-e) Number of detected HGT breakpoint pairs for different genomes at varying sequencing depths. Genomes were classified into two categories based on their maximum count of breakpoint pairs: 5-20 (d) and >20 (e). In this analysis, we excluded genomes that had fewer than 5 breakpoint pairs when the depth was over 30x. Each blue point on the graph represents a genome with a specific depth, showcasing the number of detected breakpoint pairs. The orange line represents the saturation curve. Saturation curves were obtained through regression analysis on exponential functions. (f) Number of detected HGT breakpoint pairs at different sequencing depths for genomes of different lengths. The genomes were categorized by their lengths. Red points indicate genomes with lengths shorter than 3M bp, while blue triangles represent genomes with lengths longer than 3M bp.

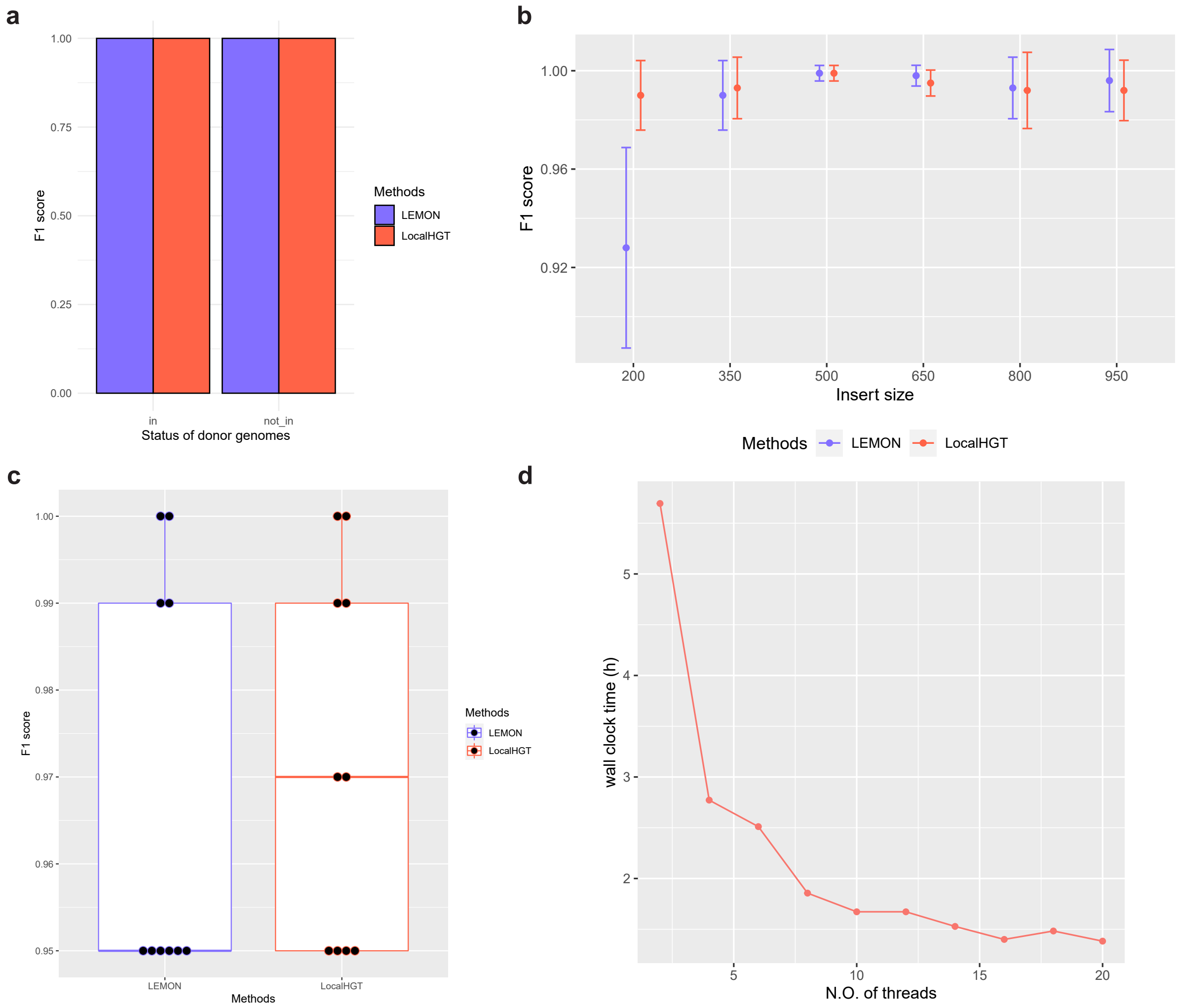

Figure S9: Evaluation of LocalHGT with various settings. (a) Evaluating LocalHGT with the presence/absence of the donor genome. 'in' represents the sample containing the donor genome, and 'not_in' means the donor genome is absent in the sample. The height of the bar shows the mean F1 score for HGT breakpoint detection. (b) Evaluation of LocalHGT with different insert sizes. We simulated samples with varying insert sizes from 200 bp to 950 bp. Ten samples were generated for each insert size. Then we compared the HGT breakpoint detection accuracy of LocalHGT and LEMON on these samples. The dot indicates the mean F1 score for HGT breakpoint detection, and the error bar represents the standard variation. (c) HGT breakpoint detection accuracy of LocalHGT and LEMON with short read length. To evaluate the accuracy of LocalHGT with short read length, we simulated paired-end reads with a length of 75 bp in ten samples. Each dot in the box represents the F1 score of HGT breakpoint detection. (d) Wall-clock time of LocalHGT with different numbers of threads. With each specific number of threads, we ran LocalHGT on the CAMI high-complexity sample three times and calculated the average wall-clock time (hours). We increased the number of threads from 2 to 20, adding 2 threads each time.

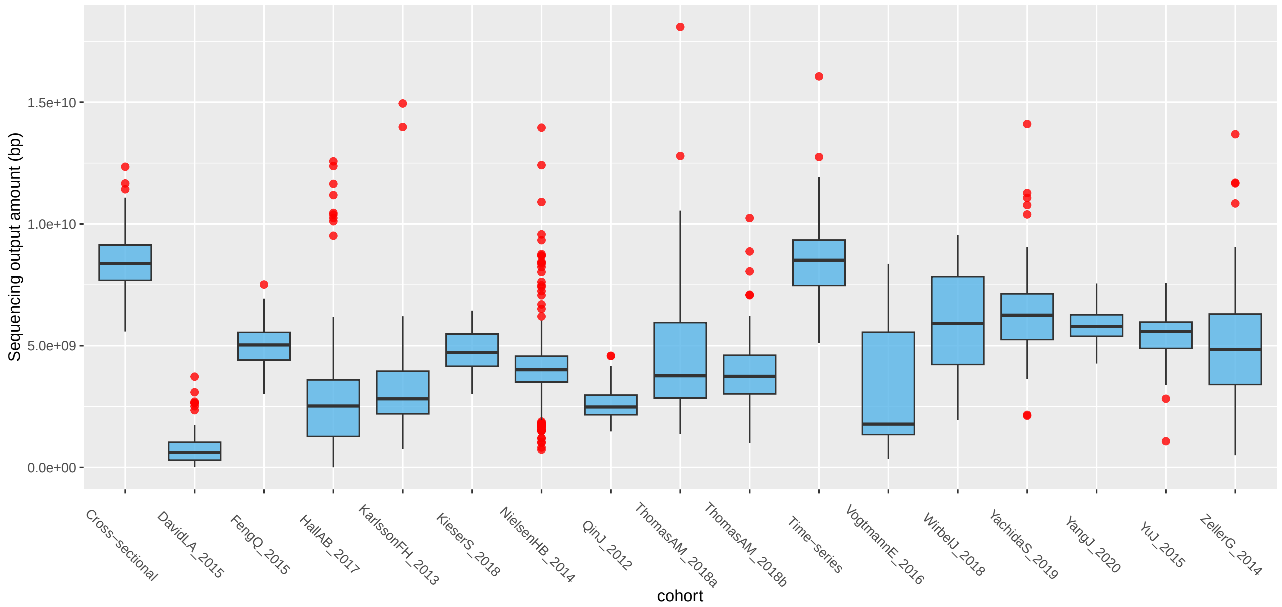

Figure S10: Sequencing output amount of different data cohorts. The sequencing output amount of each sample was quantified based on the number of base pairs present in the sequencing reads.

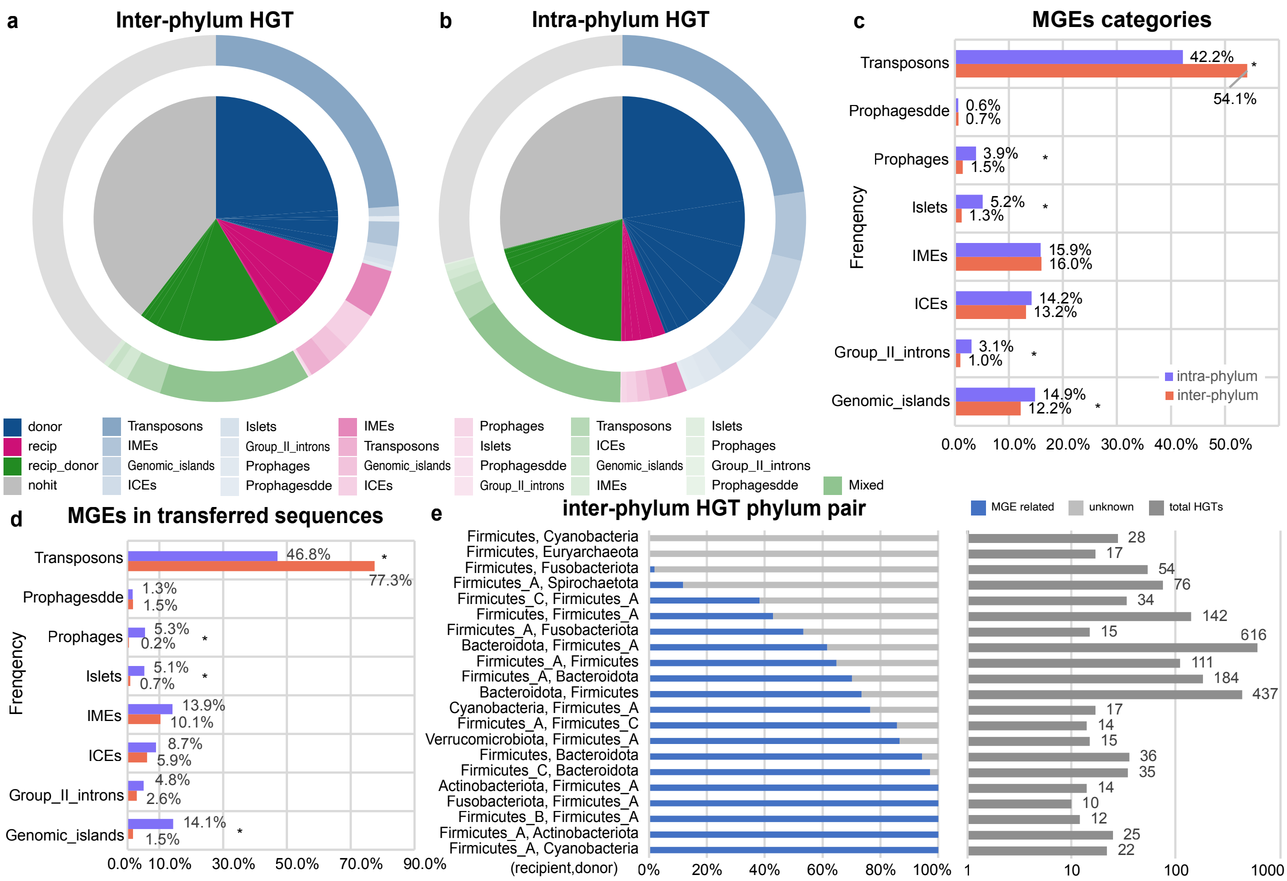

Figure S11: MGEs associated with inter-phylum and intra-phylum HGT events. (a-b) Frequencies of inter-phylum (a) and intra-phylum (b) HGT events that had associated MGEs in the donor genome ('donor'), the recipient genome ('recipient') and both genomes ('recip_donor'). The MGEs were further categorized into various categories. 'Mixed' in 'recip_donor' means MGEs in the recipent genome and the donor genome belongs to different MGEs categories. (c-d) Frequencies of MGE categories involved in HGT events (c) and present in transferred sequences (d) differ between inter-phylum (red) and intra-phylum (blue) HGTs. The symbol * indicates *P*-value $<$ 0.05. (e) Frequency of inter-phylum HGT events associated with MGEs for various phylum pairs. The right subplot illustrates the number of inter-phylum HGT events belonging to each phylum pair. The values on the right subplot are presented in a logarithmic scale.

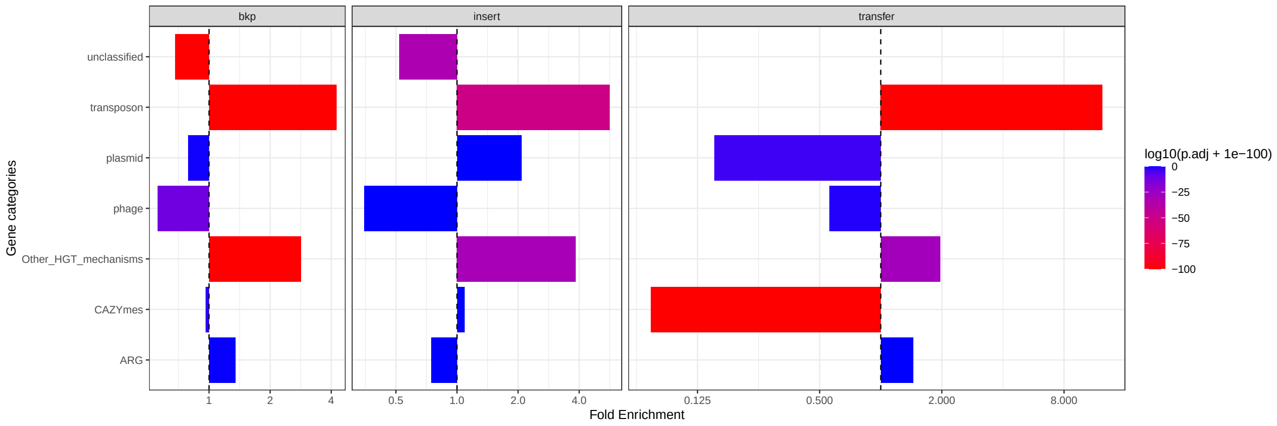

Figure S12: Gene functional classification enrichment of HGT-related genes.
The enriched and depleted gene classifications of the genes surrounding HGT 'breakpoint' (BKP), 'insert site' (insert), and 'transferred sequence' (transfer). The color indicates the *P*-value of the enrichment.

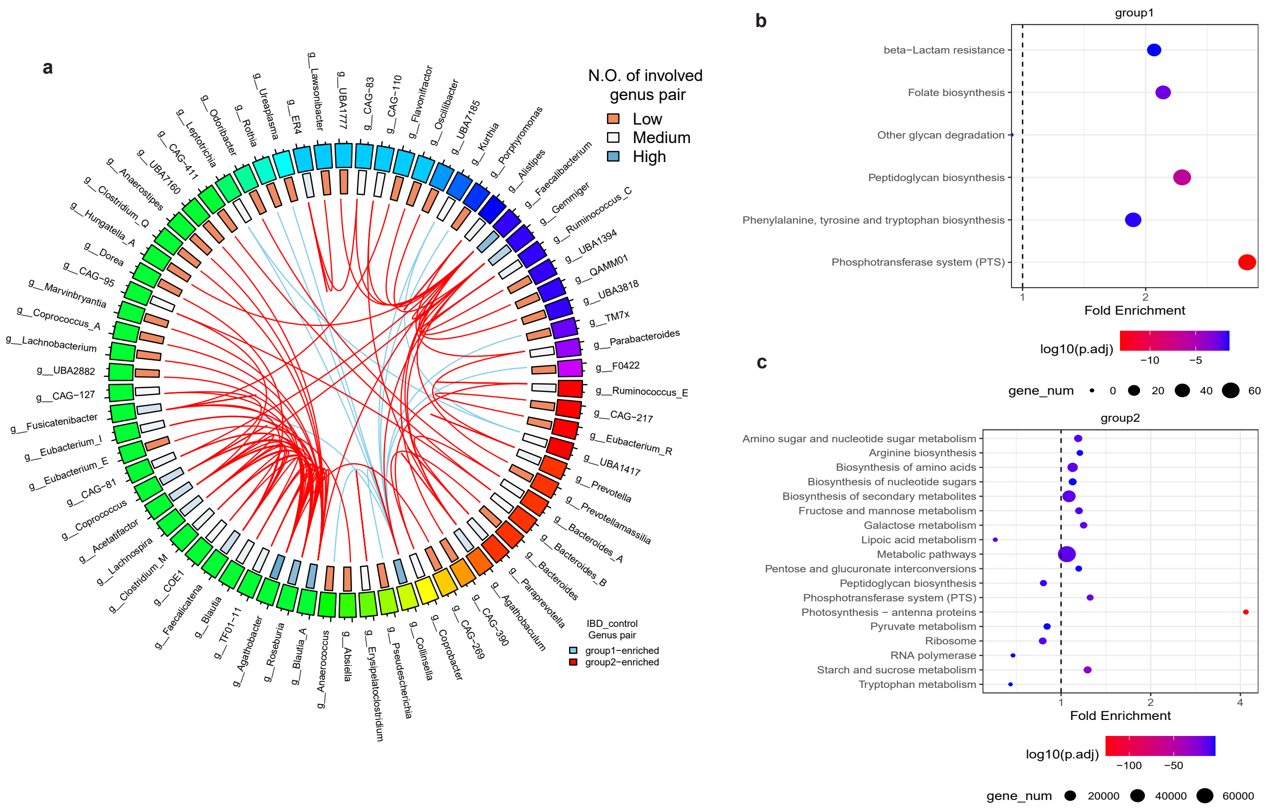

Figure S13: Association between HGT and IBD. ‘Group1’ represents IBD and ‘Group2’ represents controls. (a) The illustration of the differential genus pairs. Each cell in the first track means a genus, and the genera of the same family are marked with a same color. The gradient color in the second track represents the number of involved differential genus pairs of each genus. The inner line indicates the genus pairs. The blue line means the genus pair is enriched in IBD and red line means the genus pair is enriched in controls. (b,c) The enriched and depleted KEGG pathways in the genes surrounding HGT breakpoints of IBD-enriched (b) and control-enriched genus pairs (c).

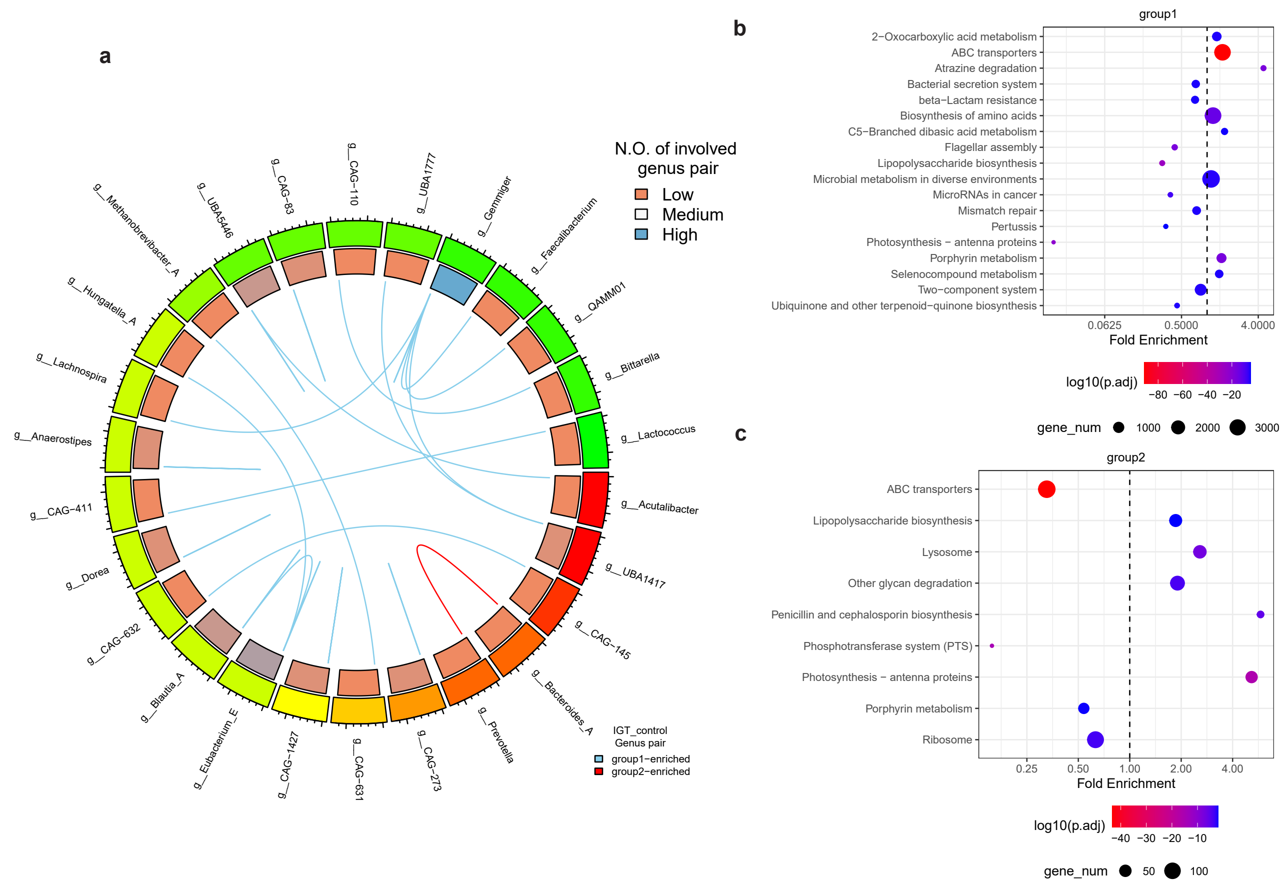

Figure S14: Association between HGT and IGT. 'Group1' represents IGT and 'Group2' represents controls. (a) The illustration of the differential genus pairs. Each cell in the first track means a genus, and the genera of the same family are marked with a same color. The gradient color in the second track represents the number of involved differential genus pairs of each genus. The inner line indicates the genus pairs. The blue line means the genus pair is enriched in IGT and red line means the genus pair is enriched in controls. (b,c) The enriched and depleted KEGG pathways in the genes surrounding HGT breakpoints of IGT-enriched (b) and control-enriched genus pairs (c).

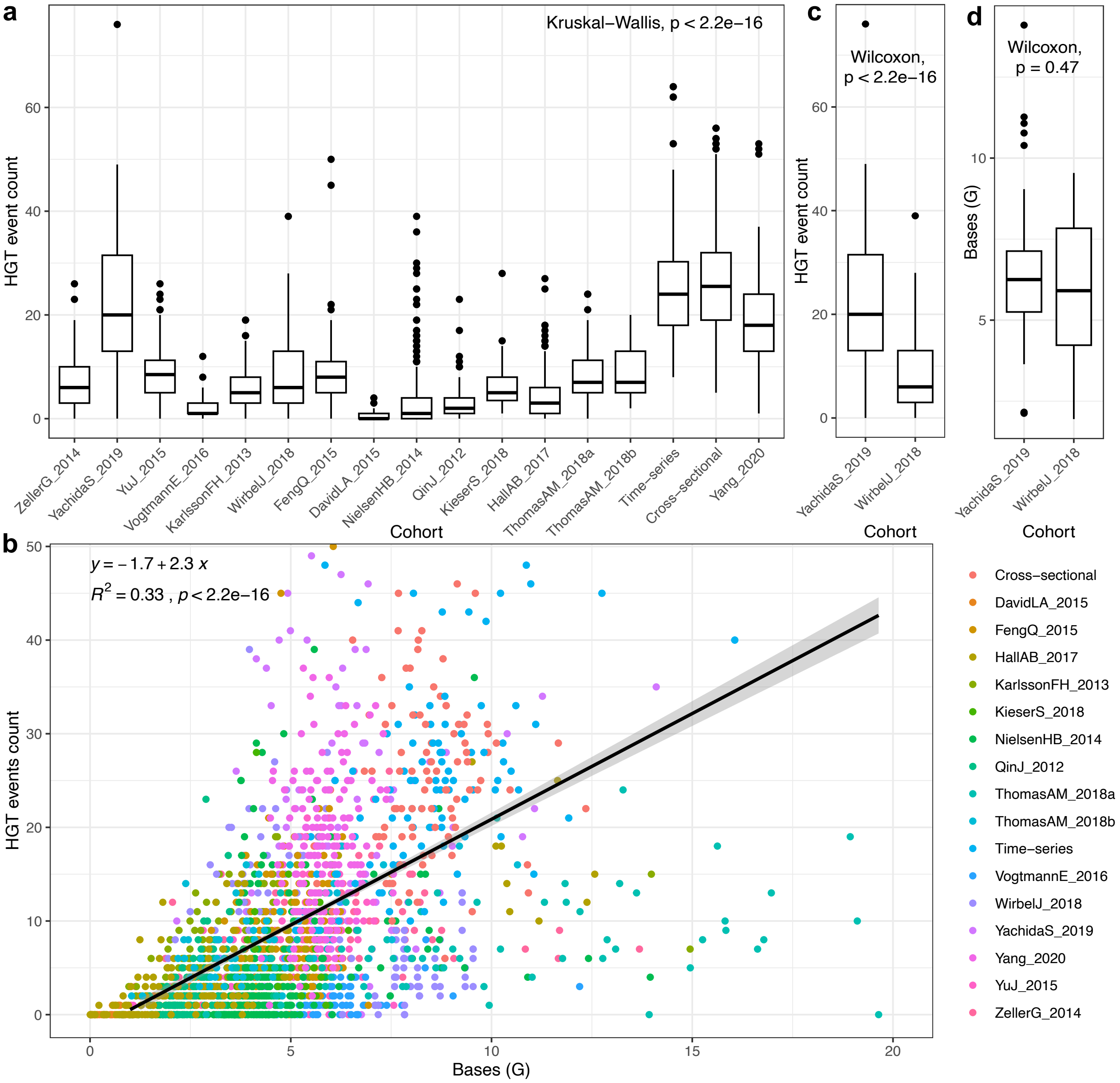

Figure S15: Investigating the impact of wet lab procedures on HGT detection. (a) Comparison of HGT event counts across all cohorts through a boxplot. The Kruskal-Wallis test was employed to assess the differences in HGT event counts across all cohorts. (b) A scatter plot depicting samples from all cohorts, with DNA base count on the x-axis and HGT event counts on the y-axis, featuring a black line representing the linear regression. The plot also includes the formula of the regression line, along with the corresponding R^2 value and *P*-value. (c) Comparison of HGT event counts between the YachidaS_2019 and WirbelJ_2018 cohorts, which underwent similar wet lab procedures. (d) Comparison of the number of DNA bases between the two cohorts. The comparison was performed using Wilcox rank sum testing.

Supplemental References

[1] Liang Chen, Na Zhao, Jiabao Cao, Xiaolin Liu, Jiayue Xu, Yue Ma, Ying Yu, Xuan Zhang, Wenhui Zhang, Xiangyu Guan, et al. Short-and long-read metagenomics expand individualized structural variations in gut microbiomes. *Nature Communications*, 13(1):3175, 2022.

[2] Heng Li. Minimap2: pairwise alignment for nucleotide sequences. *Bioinformatics*, 34(18):3094–3100, 2018.

[3] Alexandre Almeida, Stephen Nayfach, Miguel Boland, Francesco Strozzi, Martin Beracochea, Zhou Jason Shi, Katherine S Pollard, Ekaterina Sakharova, Donovan H Parks, Philip Hugenholtz, et al. A unified catalog of 204,938 reference genomes from the human gut microbiome. *Nature biotechnology*, 39(1):105–114, 2021.

[4] Benjamin Hillmann, Gabriel A Al-Ghalith, Robin R Shields-Cutler, Qiyun Zhu, Daryl M Gohl, Kenneth B Beckman, Rob Knight, and Dan Knights. Evaluating the information content of shallow shotgun metagenomics. *Msystems*, 3(6):10–1128, 2018.

[5] Derrick E Wood, Jennifer Lu, and Ben Langmead. Improved metagenomic analysis with kraken 2. *Genome biology*, 20:1–13, 2019.

[6] Shuai Wang, Yiqi Jiang, and Shuaicheng Li. Pstrain: an iterative microbial strains profiling algorithm for shotgun metagenomic sequencing data. *Bioinformatics*, 36(22-23):5499–5506, 2020.

[7] Weichun Huang, Leping Li, Jason R Myers, and Gabor T Marth. Art: a next-generation sequencing read simulator. *Bioinformatics*, 28(4):593–594, 2012.

[8] Alexander Sczyrba, Peter Hofmann, Peter Belmann, David Koslicki, Stefan Janssen, Johannes Dröge, Ivan Gregor, Stephan Majda, Jessika Fiedler, Eik Dahms, et al. Critical assessment of metagenome interpretation—a benchmark of metagenomics software. *Nature methods*, 14(11):1063–1071, 2017.

[9] Xiaofang Jiang, Andrew Brantley Hall, Ramnik J Xavier, and Eric J Alm. Comprehensive analysis of chromosomal mobile genetic elements in the gut microbiome reveals phylum-level niche-adaptive gene pools. *PloS one*, 14(12):e0223680, 2019.

[10] Louis-Charles Fortier and Ognjen Sekulovic. Importance of prophages to evolution and virulence of bacterial pathogens. *Virulence*, 4(5):354–365, 2013.

[11] FB Guo, W Wei, XL Wang, H Lin, H Ding, J Huang, and N Rao. Co-evolution of genomic islands and their bacterial hosts revealed through phylogenetic analyses of 17 groups of homologous genomic islands. *Genet. Mol. Res*, 11(4):3735–3743, 2012.

[12] Michael Karberg, Huatao Guo, Jin Zhong, Robert Coon, Jiri Perutka, and Alan M Lambowitz. Group ii introns as controllable gene targeting vectors for genetic manipulation of bacteria. *Nature biotechnology*, 19(12):1162–1167, 2001.

[13] Nicolás Toro, José Ignacio Jiménez-Zurdo, and Fernando Manuel García-Rodríguez. Bacterial group ii introns: not just splicing. *FEMS microbiology reviews*, 31(3):342–358, 2007.

[14] Aonghus Lavelle, Stéphane Nancey, Jean-Marie Reimund, David Laharie, Philippe Marteau, Xavier Treton, Matthieu Allez, Xavier Roblin, Georgia Malamut, Cyriane Oeuvray, et al. Fecal microbiota and bile acids in ibd patients undergoing screening for colorectal cancer. *Gut microbes*, 14(1):2078620, 2022.

[15] Giuseppe Lo Sasso, Lusine Khachatryan, Athanasios Kondylis, James ND Battey, Nicolas Sierro, Natalia A Danilova, Tatiana V Grigoryeva, Maria I Markelova, Dilyara R Khusnutdinova, Alexander V Laikov, et al. Inflammatory bowel disease–associated changes in the gut: focus on kazan patients. *Inflammatory bowel diseases*, 27(3):418–433, 2021.

[16] Tapan Behl, Aayush Sehgal, Madhuri Grover, Sukhbir Singh, Neelam Sharma, Saurabh Bhatia, Ahmed Al-Harrasi, Lotfi Aleya, and Simona Bungau. Uncurtaining the pivotal role of abc transporters in diabetes mellitus. *Environmental Science and Pollution Research*, 28(31):41533–41551, 2021.
